# Model-Estimated Association Between Simulated US Elementary School–Related SARS-CoV-2 Transmission, Mitigation Interventions, and Vaccine Coverage Across Local Incidence Levels

**DOI:** 10.1001/jamanetworkopen.2021.47827

**Published:** 2022-02-14

**Authors:** John Giardina, Alyssa Bilinski, Meagan C. Fitzpatrick, Emily A. Kendall, Benjamin P. Linas, Joshua Salomon, Andrea L. Ciaranello

**Affiliations:** 1 Center for Health Decision Science, Harvard T.H. Chan School of Public Health, Boston, Massachusetts; 2 Department of Health Services, Policy, and Practice, Department of Biostatistics, Brown School of Public Health, Providence, Rhode Island; 3 Center for Vaccine Development and Global Health, University of Maryland School of Medicine, Baltimore; 4 Division of Infectious Diseases, Johns Hopkins University School of Medicine, Baltimore, Maryland; 5 Boston University Schools of Medicine and Public Health, Boston Medical Center, Boston, Massachusetts; 6 Center for Health Policy and Center for Primary Care and Outcomes Research, Stanford University School of Medicine, Stanford, California; 7 Division of Infectious Disease and Medical Practice Evaluation Center, Massachusetts General Hospital, Boston

## Abstract

**Importance:**

With recent surges in COVID-19 incidence and vaccine authorization for children aged 5 to 11 years, elementary schools face decisions about requirements for masking and other mitigation measures. These decisions require explicit determination of community objectives (eg, acceptable risk level for in-school SARS-CoV-2 transmission) and quantitative estimates of the consequences of changing mitigation measures.

**Objective:**

To estimate the association between adding or removing in-school mitigation measures (eg, masks) and COVID-19 outcomes within an elementary school community at varying student vaccination and local incidence rates.

**Design, Setting, and Participants:**

This decision analytic model used an agent-based model to simulate SARS-CoV-2 transmission within a school community, with a simulated population of students, teachers and staff, and their household members (ie, immediate school community). Transmission was evaluated for a range of observed local COVID-19 incidence (0-50 cases per 100 000 residents per day, assuming 33% of all infections detected). The population used in the model reflected the mean size of a US elementary school, including 638 students and 60 educators and staff members in 6 grades with 5 classes per grade.

**Exposures:**

Variant infectiousness (representing wild-type virus, Alpha variant, and Delta variant), mitigation effectiveness (0%-100% reduction in the in-school secondary attack rate, representing increasingly intensive combinations of mitigations including masking and ventilation), and student vaccination levels were varied.

**Main Outcomes and Measures:**

The main outcomes were (1) probability of at least 1 in-school transmission per month and (2) mean increase in total infections per month among the immediate school community associated with a reduction in mitigation; multiple decision thresholds were estimated for objectives associated with each outcome. Sensitivity analyses on adult vaccination uptake, vaccination effectiveness, and testing approaches (for selected scenarios) were conducted.

**Results:**

With student vaccination coverage of 70% or less and moderate assumptions about mitigation effectiveness (eg, masking), mitigation could only be reduced when local case incidence was 14 or fewer cases per 100 000 residents per day to keep the mean additional cases associated with reducing mitigation to 5 or fewer cases per month. To keep the probability of any in-school transmission to less than 50% per month, the local case incidence would have to be 4 or fewer cases per 100 000 residents per day.

**Conclusions and Relevance:**

In this study, in-school mitigation measures (eg, masks) and student vaccinations were associated with substantial reductions in transmissions and infections, but the level of reduction varied across local incidence. These findings underscore the potential role for responsive plans that deploy mitigation strategies based on local COVID-19 incidence, vaccine uptake, and explicit consideration of community objectives.

## Key Points

**Question:**

How is COVID-19 incidence in elementary school communities associated with in-school mitigation (eg, masks), vaccination, and local incidence, and when should decision-makers add or remove mitigation measures?

**Findings:**

In this decision analytic model with a simulated population of 638 students and 60 educators and staff in an elementary school, school community incidence decreased with mitigation and vaccination and increased with local incidence. Thresholds for changing mitigation measures depended on the objective (eg, minimizing likelihood of any in-school transmission vs maintaining cases within acceptable limits).

**Meaning:**

These findings suggest that appropriate increases and decreases for in-school mitigation depend on policy makers’ goals; responsive plans, in which mitigation is deployed based on local COVID-19 incidence and vaccine uptake, may be appropriate.

## Teaser

This decision analytic model estimates the associations of adding or removing in-school mitigation measures with COVID-19 outcomes within an elementary school community at varying student vaccination and local incidence rates.

## Introduction

To balance the educational and social and emotional benefits of in-person education with concerns about SARS-CoV-2 transmission in school settings, the US Centers for Disease Control and Prevention (CDC) recommends using a layered mitigation approach in kindergarten to 12th grade (K-12) schools. Some components of this approach include vaccination for all eligible students and educators and staff, improved ventilation, and indoor masking regardless of vaccination status.^1^ Individual states and school districts make local decisions about whether and how to incorporate these recommendations, and requirements for indoor masking have particularly generated debate.^2^ In communities with high vaccination rates and low COVID-19 incidence, or where masking is less widely accepted, many schools are considering removing masks and other elements of mitigation.^3,4^

While multiple studies indicate that masks are effective at mitigating the transmission of upper respiratory viruses,^5,6,7,8,9,10^ they are generally viewed as a temporary measure.^11,12^ Masks are physiologically safe, but there is limited data on the impact of mask-wearing on learning and social and emotional development, especially for younger children, students with special learning needs, and English language learners.^9,13^ With the availability of vaccines for all US residents aged 5 years and older, many public health experts have called for “off-ramps” and “on-ramps” that use available public health data to inform decisions about when to remove or reinstate masking and other mitigation measures.^11,12,14,15^

Establishing these off-ramps and on-ramps requires decision-makers to be explicit about the objectives they seek to achieve, which in turn necessitates a quantitative estimate of the epidemiologic consequences of adding or removing mitigation. We used a previously published simulation model of SARS-CoV-2 transmission within an elementary school community to generate estimates across a range of potential assumptions about intervention effectiveness, student vaccine coverage, and observed local COVID-19 incidence.^16^ We evaluated decision thresholds for multiple objectives to support decision-makers across different contexts.

## Methods

### Modeled Population and Model Structure

We simulated an elementary school with 638 students in 30 separate classes and 60 educators and staff. Household members included 2 adults in each student household (with sibling students grouped in the same household) and 1 additional adult in each educator and staff household. The study adheres to the Consolidated Health Economic Evaluation Reporting Standards (CHEERS) reporting guidelines^17^ and was designated not human participant research by the Mass General Brigham institutional review board.

The model simulates infection dynamics within the immediate school community (students, educators and staff, and family members) and tracks infections over 30 days. At school, students, educators, and staff interact: within classrooms, during so-called specials classes (eg, related arts), and through random contacts. Outside of school, students and educator and staff interact with household members and other families (simulating social interactions or shared childcare). SARS-CoV-2 is introduced to the immediate school community at a rate proportional to the observed incidence rate for the wider local community (after accounting for an assumed case ascertainment rate).

Transmissions from infected people are modeled as a function of the age (student vs adult) of the infected individual and contact, vaccination status of the contact, and duration and location of exposure, with the latent and infectious periods drawn from distributions with means of 3.5 and 5 days, respectively.^18,19,20,21,22,23^ In-school mitigation measures are simulated as a relative risk reduction on in-school transmission risk. Symptomatic students, educators, and staff with a clinical (vs subclinical) infection are offered diagnostic testing; for selected scenarios, we included weekly polymerase chain reaction screening offered to all students, educators, and staff. People identified with SARS-CoV-2 isolate for 7 days, and in-school contacts quarantine for 7 days. (We assumed all members of a classroom are in-school contacts). Additional details on the model structure are in eMethods 1 in the Supplement and the article by Bilinski et al.^16^

### Input Parameters

Selected input parameters are listed in the Table, eMethods 1 and eMethods 2 in the Supplement.^16,18,19,20,21,22,23,24,25,26,27,28,29,30,31,32,33,34,35,36,37,38,39,40,41,42,43,44,45,46,47,48,49,50^ Bilinski et al^16^ describe other model input.

**Table.** Selected Input Parameters for Agent-Based Dynamic Transmission Model of 30-Day SARS-CoV-2 Outcomes in Elementary Schools

Parameter	Values	Source
Full day in-school symptomatic adult-to-adult secondary attack rate (unmitigated)		
Wild-type	2.0%	Bilinski et al,^16^ 2021; Doyle et al,^24^ 2021^a^
Alpha	3.5%	Davies et al,^25^ 2021^a^
Delta	7.0%	Singanayagam et al,^26^ 2021; Dougherty et al,^27^ 2021; National Centre for Immunisation Research and Surveillance,^28^ 2021^a^
Attack rate multipliers by location and duration of contact (relative to full day in-school contact)		
At-home contacts	2^b^	Assumption based on documented increased attack rates in the home (Thompson et al,^29^ 2021) and increased time in close proximity
Brief contacts at school (random and specials classes)	0.125^b^	Assumed to last 1 period out of an 8-period day, with infection risk proportional to time
Brief contacts at school (staff-staff contacts)	0.25^b^	Assumed to last 1 period out of an 8-period day, but with higher risk from closer proximity (eg, break room)
Contacts between households (eg, childcare)	1	Assumption; in-school mitigation measures are not applied to these contacts
Infectiousness (relative to symptomatic adults)		
Student (in-school and asymptomatic at-home)	0.5^b^	Literature review and calibration from Bilinski et al,^16^ 2021
Asymptomatic adult	0.5^b^	Byambasuren et al,^30^ 2020; He et al,^31^ 2020
Student (symptomatic at-home)	1	Paul et al,^32^ 2021
Overdispersion multiplier (for adults)	Lognormal distribution (0.84, 0.3)/0.84^b^	Kerr et al,^22^ 2020; Endo et al,^33^ 2020
Susceptibility (relative to adults)		
Student	0.5^b^	Literature review and calibration from Bilinski et al,^16^ 2021
Length of latent and incubation periods and infection (days)		
Time from exposure to infectious (latent period)	Maximum of gamma distribution (5.8, 0.95) minus normal distribution (2, 0.4); 1^b^	Lauer et al,^18^ 2020; He et al,^19^ 2020; Li et al,^20^ 2020; Gatto et al,^21^ 2020
Time from exposure to symptoms (if symptoms occur) (incubation period)	Gamma distribution (5.8, 0.95)^b^	Lauer et al,^18^ 2020; Li et al,^20^ 2020
Duration of infectious period	Lognormal distribution (5, 2)^b^	Li et al,^20^ 2020; Kerr et al,^22^ 2020; He et al,^19^ 2020; Firth et al,^23^ 2020^c^
Probability clinical/symptomatic infection		
Probability of asymptomatic infection		
Student	0.4^b^	Fontanet et al,^34^ 2021; Stein-Zamir et al,^35^ 2020
Adult	0.2^b^	Byambasuren et al,^30^ 2020
Probability of subclinical infection, including asymptomatic		
Student	0.8^b^	Han et al,^36^ 2021
Adult	0.4^b^	Upper bound of estimate from Byambasuren et al,^30^ 2020
Polymerase chain reaction test characteristics		
Sensitivity (during infectious period)	0.9 (asymptomatic testing); 1 (symptomatic testing)^b^	Atkeson et al,^37^ 2021; Larremore et al,^38^ 2021; Cevik et al,^39^ 2021; Wyllie et al,^40^ 2020; Kojima et al,^41^ 2021
Test turnaround time, d	1^b^	Assumption
Weekly screening parameters		
Testing uptake (fraction of school screened each week)	90%^b^	Assumption
Testing day	Monday^b^	Assumption
Hospitalization risk after SARS-CoV-2 infection		
Student (unvaccinated)	0.1%	US Centers for Disease Control and Prevention,^42^ 2021; Delahoy et al,^43^ 2021^a^
Adult (unvaccinated)	2.4%	US Centers for Disease Control and Prevention,^42^ 2021^a^
All (vaccinated)	0%	Rosenberg et al,^44^ 2021^a^
Vaccine uptake		
Student	0%, 25%, 50%, and 70% (base case); 90% (sensitivity analysis)	Assumption
Adult	70% (base case); 50% and 90% (sensitivity analysis)	US Centers for Disease Control and Prevention,^45^ 2021
Vaccine effectiveness		
All individuals	70% reduction in infection risk (base case); 25%, 50%, and 90% (sensitivity analysis)	Rosenberg et al,^46^ 2021; Keehner et al,^47^ 2021; Fowlkes et al,^48^ 2021; Puranik et al,^49^ 2021; Zeng et al,^50^ 2021^a^
Risk of exposure in wider local community		
Observed local incidence rate	0-50 cases per 100 000 residents per d	Assumption
Actual incidence of infections within immediate school community sourced from wider local community	3 × observed local incidence rate	Assumption

^a^ eMethods 1 in the Supplement includes an explanation of how these parameters were derived from the listed sources.

^b^ Baseline parameter from Bilinski, et al.^16^

^c^ This value was set to match the generation time implied by observed estimates of the serial interval and presymptomatic transmission, without assuming waning infectiousness.

#### Infectiousness and Hospitalization Risk

We assumed full-day symptomatic adult-to-adult in-school “secondary attack rates” (SARs) of 2%, 3.5%, and 7% per day for the wild-type virus, Alpha variant, and Delta variant, respectively (eMethods 1 in the Supplement). The full-day SAR is defined as the proportion of susceptible adults exposed to a symptomatic adult index case who acquire SARS-CoV-2 infection per day of contact in the absence of mitigation. Wild-type and Alpha variants are included to provide results against which schools can compare observed data from the 2020 to 2021 academic year. We assumed that elementary students were half as infectious as adults in schools and equally infectious in household settings.^16,32^

Using infection fatality rate and in-hospital mortality rates provided by the CDC for use in COVID-19 models and relative hospitalization rates in different age groups, we assumed hospitalization risks among unvaccinated students and adults (aged 18 to 49 years) with COVID-19 of 0.1% and 2.4%, respectively, and a negligible risk among vaccinated individuals younger than 49 years (eMethods 1 in the Supplement).^42,43,44^

#### Vaccine Uptake and Effectiveness

In the base case, we assumed 70% uptake of 2-dose vaccination among adults (including educators, staff, and household members), reflecting US national data,^45^ along with 4 potential scenarios of student vaccine uptake (0%, 25%, 50%, and 70%). In sensitivity analyses, we examine 50% adult vaccine uptake and a scenario in which both adults and students have 90% uptake. Given recent observational data on waning vaccine effectiveness, we assumed a base case of 70% vaccine effectiveness,^46,47,48,49,50^ along with sensitivity analyses at 90%, 50%, and 25% effectiveness (eMethods 1 in the Supplement).

#### Mitigation Effectiveness

In the absence of data on the independent impact of individual mitigation measures on transmission, we estimated wide ranges for the effectiveness of 3 packages of interventions: simple ventilation and handwashing (group A; 20%-40% effective); group A plus universal masking (group B; 60%-80% effective); and full implementation of CDC-recommended measures^1^ from 2020 to 2021 (eg, group B plus physical distancing of 3-6 feet when masked and >6 when unmasked, daily cleaning of surfaces, restrictions on shared items, and cohorting of students) (group C; 90%-100% effective). Group A effectiveness was based on the results of available airflow and air quality studies^51,52^; group B effectiveness was based on both clinical as well as droplet and/or aerosol studies evaluating masking effectiveness^5,6,7,8,9,10^ and a study evaluating the combination of masking and ventilation in a controlled environment^53^; and group C effectiveness was based on observed risk of in-school transmission (0%-3% over the full infectious period) in schools implementing a full suite of mitigation measures in 2020 to 2021 (eMethods 2 in the Supplement).^54,55,56^ The estimates for A and B are based on limited available data and remain highly uncertain; approximate ranges are used to understand the potential consequences of moving between mitigation approaches, and schools may define their specific values within each range based on local degree of implementation.

#### Simulated Scenarios

The base case included scenarios reflecting wild-type virus, Alpha variant, and Delta variant, different student vaccination coverage (0%, 25%, 50%, and 70% coverage), and 70% adult vaccination uptake. For each variant, we ran the model across a range of observed local incidence levels (0-50 cases per 100 000 residents per day, assumed 33% of cases observed) and in-school mitigation effectiveness (0%-100% reduction to in-school attack rate). To present smoothed results across these continuous ranges and manage the relatively high degree of model stochasticity from discrete model output, we constructed a regression-based meta-model from the raw model output to estimate the outcomes of interest (eMethods 3 in the Supplement).^57^ We conducted the sensitivity analyses discussed previously only on the Delta variant scenarios, as these are most relevant for current decision-making.

#### Outcomes and Decision Thresholds

We evaluated 2 primary outcomes over a 30-day period: (1) probability of any in-school SARS-CoV-2 transmission at each level of mitigation effectiveness and (2) mean increase in total infections among students, educators, staff, and their household members (ie, the immediate school community) associated with moving from more to less intensive mitigation measures (eg, unmasking). For the second outcome, we projected the increase in cases associated with each of 3 discrete changes in mitigation effectiveness, reflecting possible values of the difference between the A and B mitigation scenarios described previously, ie, a change from 60% to 40% mitigation effectiveness (between inner bounds of the respective effectiveness estimates); from 70% to 30% effectiveness (between midpoints); and from 80% to 20% effectiveness (between outer bounds). We identified the observed local incidence thresholds at which policy makers might add or remove mitigation interventions for objectives tied to these outcomes: (1) keeping the monthly probability of in-school transmission less than 25%, 50%, or 75% or (2) keeping the number of cases added to the immediate school community by removing mitigation fewer than 3, 5, or 10 cases per month.

In addition to these primary outcomes, we also evaluated the approximate number of additional hospitalizations that would result from shifting from more to less intensive mitigation by applying the approximate hospitalization risks in the Table to the second primary outcome. We then calculated local incidence thresholds for the objectives of keeping additional hospitalizations less than 1, 3, or 5 hospitalizations per 100 000 individuals in the immediate school community per month.

### Statistical Analysis

The model and all analyses were implemented in R version 4.0.2 (R Project for Statistical Computing),^58^ and the replication code is publicly available.^59^ Rather than conducting traditional statistical tests, which are not appropriate for this type of model-based analysis, we assessed the variability in the outcomes using the sensitivity analyses described previously.

## Results

**Figure 1.**
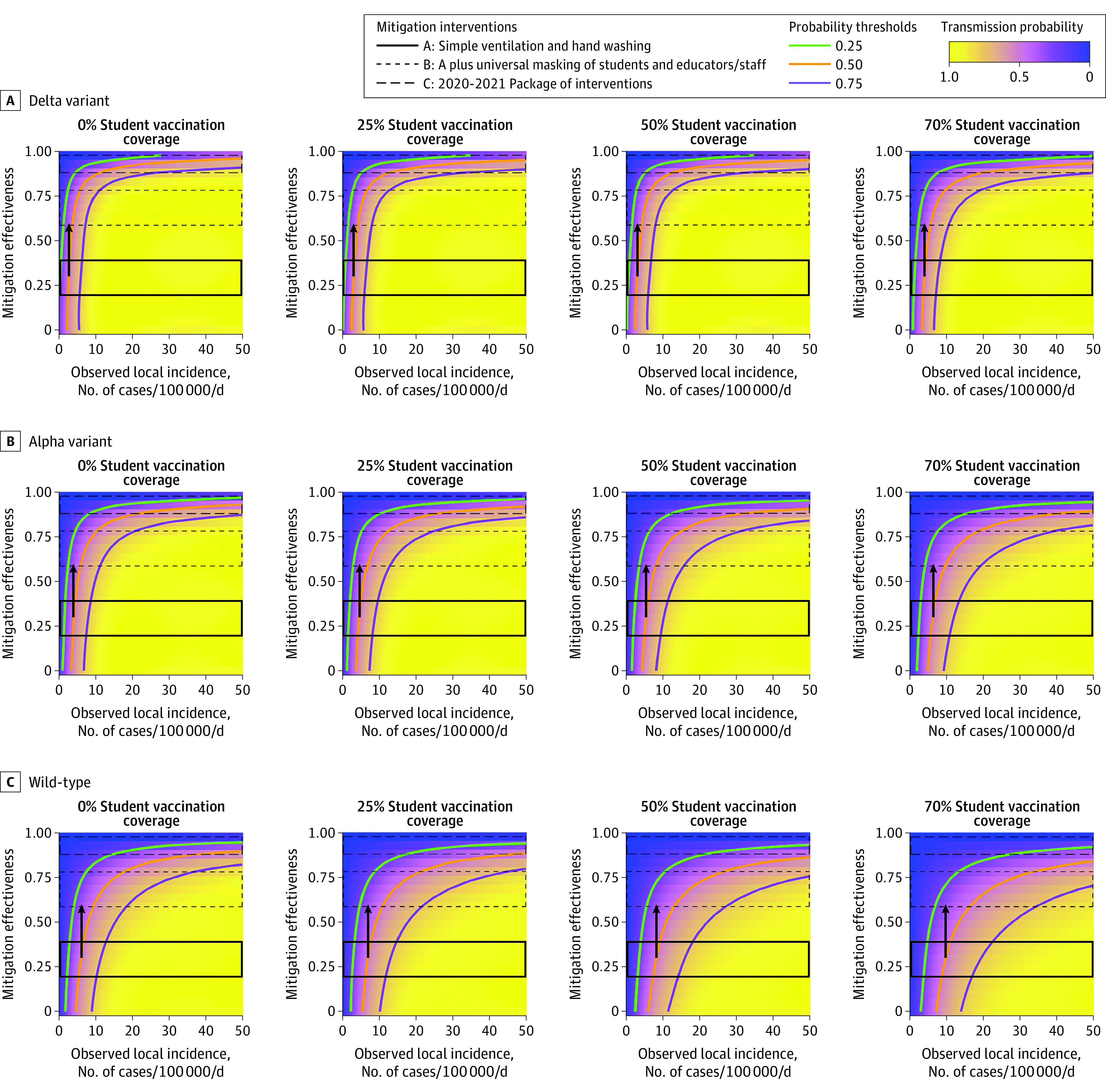
Model-Estimated Probability of at Least 1 In-School SARS-CoV-2 Transmission Over 30 Days in a Simulated Elementary School Setting Panels reflect decreasingly transmissible variants from top to bottom and increasing student vaccination coverage from left to right. Bands of mitigation effectiveness reflect approximate assumptions for the A, B, and C mitigation intervention scenarios described in the Methods section. The contour lines represent thresholds for different probability levels; probabilities are lower than the threshold above the contour line and higher below it. The arrow indicates the local COVID-19 incidence rate at which a school might opt to move to the next more intensive mitigation strategy at a baseline of 30% effectiveness, if the objective is to maintain a probability of the 1 in-school transmission per month at less than 50%. Adult vaccination coverage is assumed to be 70% in all scenarios.

**Figure 2.**
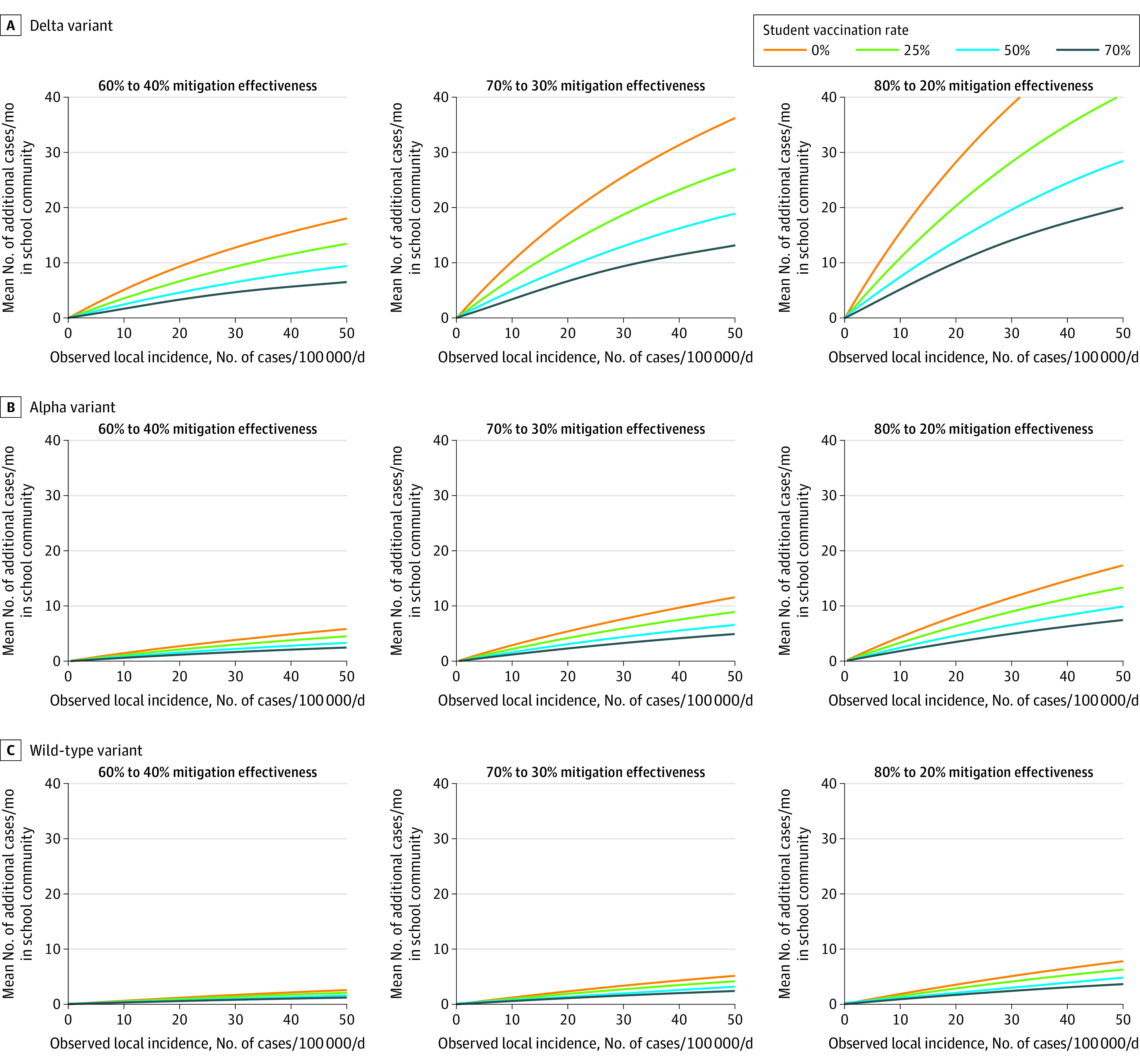
Model-Estimated Mean Number of Additional Cases Over 30 Days in the Immediate School Community Associated With Reductions in Mitigation Effectiveness in the Simulated Elementary School Setting Panels reflect decreasingly transmissible variants from top to bottom, and larger differences in effectiveness between intensive and less intensive mitigation measures from left to right. The changes in mitigation effectiveness reflect the midpoints or bounds of the A and B mitigation scenarios presented in Figure 1: 60% to 40% mitigation effectiveness (smaller effectiveness decrease); 70% to 30% effectiveness (moderate effectiveness decrease); and 80% to 20% effectiveness (larger effectiveness decrease). Adult vaccination coverage is assumed to be 70% in all scenarios.

**Figure 3.**
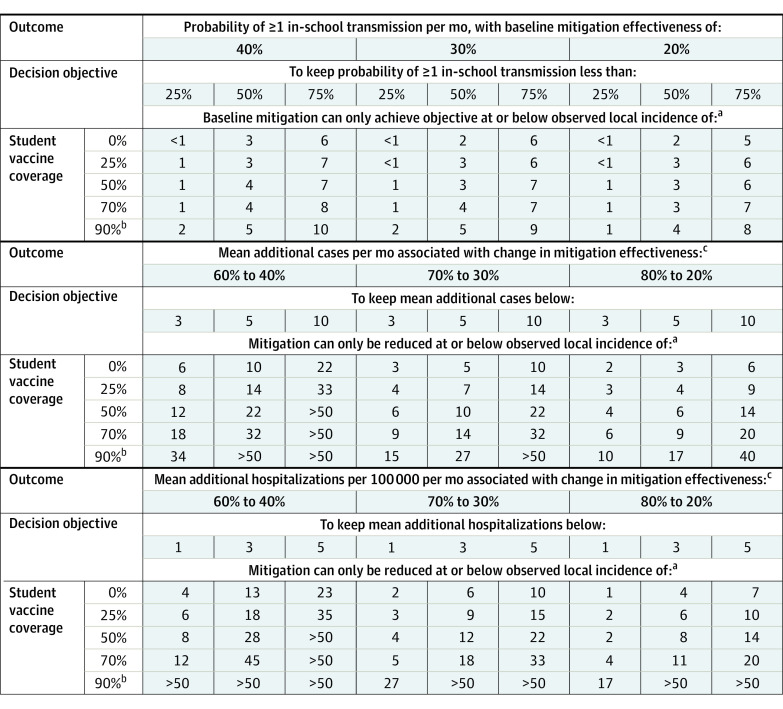
Observed Local Incidence Decision Thresholds for the Delta Variant Baseline Scenario Units of observed local incidence thresholds are cases per 100 000 residents per day. It was assumed that 33% of all actual cases are observed. ^a^If observed local incidence is above these thresholds, additional mitigation measures beyond baseline will be needed to achieve each objective (eg, keep probability of at least 1 in-school transmission per month below 50%). ^b^The Delta baseline scenario presented in this table reflects 70% adult vaccination coverage, 70% vaccine effectiveness, and no weekly screening, except for the 90% student vaccination rows, which reflect 90% adult vaccination coverage (since it is assumed adult coverage will always be at least as high as student coverage). ^c^Only includes estimated mean additional cases and hospitalizations in the immediate school community (students, teachers, staff, and household members). The potential for additional cases in the wider community stemming from in-school transmission was not modeled.

Over 30 days in the simulated elementary school, all outcomes (probability of at least 1 in-school SARS-CoV-2 transmission and the additional cases and hospitalizations associated with decreased mitigation) were substantially higher with the Delta variant and with increased local incidence and lower with increased mitigation effectiveness and higher student vaccination uptake (Figure 1 and Figure 2; eFigure 1 in the Supplement). The local incidence decision thresholds associated with meeting different objectives based on these outcomes (eg, keeping risk of in-school transmission <50%) varied across the different scenarios (Figure 3).

### Probability of In-School Transmission

With the Delta variant and 0% student vaccination, if removing masks (or other mitigation measures) was associated with a decrease in mitigation effectiveness to 30% (mitigation group A midpoint), decision-makers who seek to keep the monthly probability of in-school transmission less than 50% could remove masks at or below an observed local incidence of approximately 2 cases per 100 000 residents per day (Figure 1A). With student vaccination rates of 25%, 50%, or 70%, this threshold changed minimally to 3 to 4 cases per 100 000 residents per day (Figure 1A). Thresholds for keeping transmission probability less than 25% and less than 75% are presented in Figure 3 (for the Delta scenario) and in the Supplement for Alpha and wild-type scenarios (eTable 1 and eTable 2 in the Supplement).

### Additional Cases Associated With Mitigation Effectiveness Reduction

With the Delta variant and 0% student vaccination, if unmasking (or removing other mitigation measures) is associated with a decrease in mitigation effectiveness from 70% (group B midpoint) to 30% (group A midpoint), decision-makers who seek to keep the number of additional infections associated with removing mitigation (eg, masks) fewer than 5 per month in the immediate school community could remove masks at or below a local incidence of approximately 5 cases per 100 000 residents per day (Figure 2A). With student vaccination rates of 25%, 50%, or 70%, this threshold changed to 7, 10, or 14 cases per 100 000 residents per day, respectively (Figure 2A). If the consequences of removing masks were smaller (eg, a 60% to 40% decreases in effectiveness), these thresholds would be higher (10-32 cases per 100 000 residents per day) (Figure 2). Thresholds for keeping additional cases less than 3 or 10 infections per month are presented in Figure 3 (for the Delta scenario) and in the Supplement for the Alpha and wild-type scenarios (eTable 1 and eTable 2 in the Supplement).

### Additional Hospitalizations Associated With Mitigation Effectiveness Reduction

The rate of additional hospitalizations associated with decreases in mitigation effectiveness mirrored the additional cases and had a similar association with local incidence and student vaccination coverage (eFigure 1 in the Supplement). The local incidence thresholds required to keep the number of additional hospitalizations from mitigation reductions less than 1 per 100 000 individuals in the immediate school community per month were 12 or fewer cases per 100 000 residents per day across a range of student vaccination and mitigation effectiveness values, except with 90% vaccination for both students and adults (Figure 3). The thresholds were higher for an objective of keeping additional hospitalizations fewer than 5 per 100 000 individuals in the immediate school community per month, although still 15 or fewer cases per 100 000 residents per day for the larger changes in mitigation effectiveness (eg, 70% to 30%) with a student vaccination rate of 50% or less.

### Sensitivity Analyses

**Figure 4.**
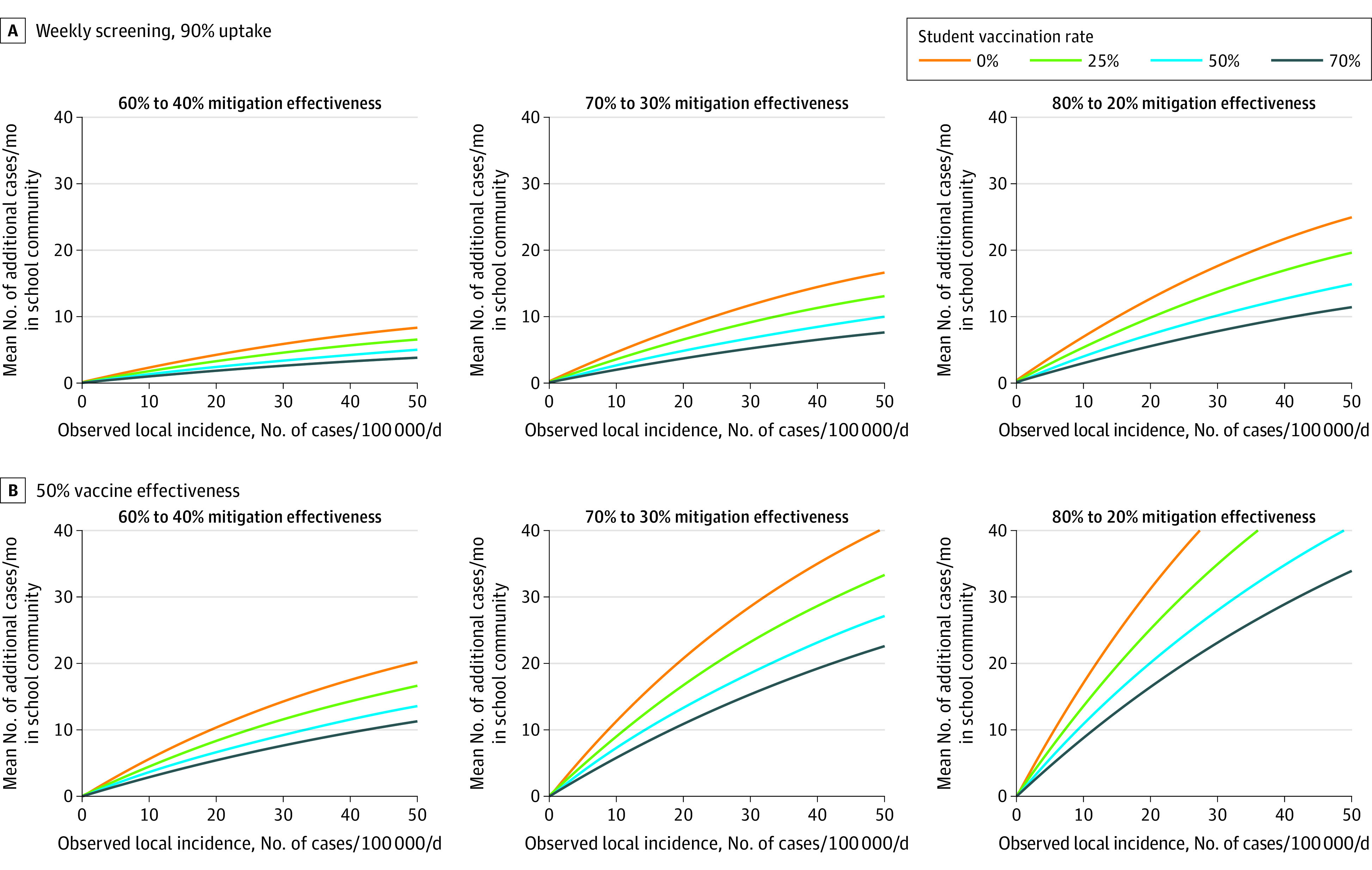
Weekly Screening and Vaccine Effectiveness Sensitivity Analyses for the Mean Number of Additional Cases Over 30 Days in the Immediate School Community Associated With Reductions in Mitigation Effectiveness in the Simulated Elementary School Setting A, This scenario is for the Delta variant, with weekly in-school screening (90% uptake) and 70% vaccine effectiveness. B, This scenario is for the Delta variant, with 50% vaccine effectiveness and only diagnostic testing. Adult vaccination coverage is assumed to be 70% in both scenarios. Panels reflect larger differences in effectiveness between intensive and less intensive mitigation measures from left to right.

When adding weekly screening of students, educators, and staff in the Delta variant scenarios, the additional cases associated with changes in mitigation effectiveness decreased substantially (Figure 4A). Assuming a decrease in mitigation effectiveness from 70% to 30%, a 50% student vaccination rate, and a goal of fewer than 5 additional cases per month in the immediate school community, decision-makers could remove mitigation at or below a local incidence of approximately 21 cases per 100 000 residents per day when weekly screening is implemented, compared with 10 cases per 100 000 residents per day with only diagnostic testing (Figure 4A, eTable 4 in the Supplement). Similarly, the probability of at least 1 in-school transmission per month decreases with the implementation of weekly screening, although the changes in decision thresholds are less stark (eFigure 3 and eTable 4 in the Supplement). The 50% and 25% vaccine effectiveness analyses (Figure 4B; eFigure 4, eFigure 5, eTable 5, and eTable 6 in the Supplement) showed increased transmission and smaller changes in the decision thresholds across student vaccination coverage compared with the 70% and 90% effectiveness analyses (Figure 1, Figure 2, Figure 3, and Figure 4; eFigure 6 and eTable 7 in the Supplement). Higher vaccination coverage in both adults and students substantially increased the local incidence thresholds (Figure 3), while lower adult vaccine coverage (ie, 50%) only moderately changed model-estimated decision thresholds, aside from the additional hospitalization objectives. The hospitalization results were sensitive to the adult vaccination rate given that unvaccinated hospitalization risk is highest in adults and we assumed complete vaccine protection against hospitalization (a conservative assumption regarding the consequences of unmasking) (eFigure 2 and eTable 3 in the Supplement).

## Discussion

We used a previously published agent-based dynamic transmission model to examine the association between vaccine uptake and effectiveness, in-school mitigation measures including masking, observed local COVID-19 incidence, and SARS-CoV-2 transmissions in an elementary school community. In order to inform ongoing decisions about masking and other measures in schools, we identified thresholds of observed local COVID-19 incidence at which decision-makers might choose to increase or decrease mitigation measures, depending on their objectives. There were 4 key findings.

First, the local incidence thresholds for adding or removing mitigation (on-ramps and off-ramps) depend on the objective that the school community seeks to achieve. When the objective is to minimize the probability of any in-school transmission, thresholds are much lower than when the objective is to keep the number of additional cases less than a given level (Figure 3). This result is intuitive, but the model provides a sense of the magnitude of this difference. Additionally, many incidence thresholds identified in this analysis are low relative to historic and current COVID-19 incidence in many districts across the United States, suggesting that even with high rates of vaccination, depending on their goals, communities may continue to find value in measures such as masking and ventilation until incidence decreases.

Second, these on-ramps and off-ramps are highly dependent on the effectiveness of each type of mitigation, which can vary across contexts and individual school settings. We evaluated a wide range of effectiveness: 20% to 40% risk reduction for simple ventilation and handwashing, 60% to 80% for ventilation and handwashing plus universal indoor masking, and 90% to 100% for the full multilayered mitigation packages often used in 2020 to 2021. Data on these measures are limited, and these ranges are uncertain; schools may be able to assess where they fall within these ranges based on adherence to past mitigation measures and the resources available. Screening of asymptomatic students, educators, and staff may be another tool to support more permissive off-ramps when unmasking is strongly desired. Weekly screening decreased the additional modeled cases associated with mitigation relaxation compared with only diagnostic testing (Figure 4A), approximately doubling the local incidence thresholds for removing other mitigation measures (eTable 4 in the Supplement), but schools need to weigh the cost of screening against these benefits. Weekly screening after unmasking may also provide valuable information about the consequences of this change in an individual school.

Third, student vaccination coverage was associated with a very substantial shift in incidence-based thresholds; less intensive in-school mitigation measures are needed to maintain lower transmission as student vaccination rates increase (Figure 3). The incidence-based thresholds were also sensitive to vaccine effectiveness. The higher modeled values (eg, 90%) may more accurately reflect recent vaccination for children (before waning vaccine effectiveness occurs)^60^ and/or booster vaccinations for adults^61^ with the Delta variant, and the lower values (eg, 25% and 50%) may reflect values in the future, with further waning or new variants, including omicron (eTables 5-7 in the Supplement).^62^ Importantly, substantial racial and economic disparities are quickly emerging in elementary student vaccination rates, mirroring these disparities in adults.^63,64^ These results demonstrate that efforts to ensure equitable access to accurate information, trustworthy messengers, and convenient vaccination sites will be critical to ensuring equitable application or relaxation of mitigation measures in schools.

Fourth, many policy makers have suggested that the objective of COVID-19 policies should be reducing hospitalizations and deaths, rather than numbers of infections or reported cases, noting that widespread availability of vaccination will reduce morbidity and mortality when infections occur.^44,65^ Although our approach to estimating hospitalization rates is approximate, it provides insight into the order of magnitude of potential hospitalizations resulting from different levels of mitigation effectiveness. To achieve even a fairly permissive objective of avoiding 5 additional hospitalizations per 100 000 individuals per month, some scenarios permit unmasking only at incidence thresholds well below 15 observed cases per 100 000 residents per day (if removing mitigation is associated with moderate or large decreases in effectiveness, with low student vaccination uptake). In scenarios with high student vaccination rates or smaller incremental mitigation effectiveness, unmasking could achieve this goal at high levels of local incidence (ie, >25 cases per 100 000 per day).

### Limitations

These results should be interpreted in the context of model limitations. First, several key data inputs were highly uncertain, including the effectiveness of individual mitigation interventions, proportions of all SARS-CoV-2 infections that are observed and reported, and hospitalization risks. To account for this uncertainty, we presented results across a range of mitigation effectiveness assumptions; incidence-based thresholds can be adjusted to reflect different proportions detected through simple multiplication (eg, to convert base-case assumption of 33% detection to 50% detection, incidence thresholds can be multiplied by 1.5); and the hospitalization rate objectives (eg, keep additional hospitalizations below 5 per 100 000 individuals per month) can be multiplied by similar conversion factors. COVID-19 incidence data at the most local level available (eg, school or city or town), including data from high-uptake asymptomatic screening, could provide the best information to inform the connection between observed and actual case counts. Additionally, this analysis focused on students, educators, staff, and their household members; additional downstream effects in the nonschool community are not captured (eg, infections from students to family outside the immediate household), which is especially relevant for the hospitalization rate results, because downstream infections in older individuals are more likely to result in hospitalizations compared with those in the relatively younger immediate school community.

## Conclusions

In this modeling study of a simulated elementary school and the risks of in-school SARS-CoV-2 transmission, we found that the risks of transmission and resulting infections among students, educators, staff, and their household members are high when a highly infectious variant predominates and students are unvaccinated. Mitigation measures or vaccinations for students substantially reduced these modeled risks. Appropriate on-ramps and off-ramps for in-school mitigation depend on the objectives that policy makers seek to achieve. These findings provide a framework for responsive plans in which mitigation is deployed based on local COVID-19 incidence and vaccine uptake. For evidence-based COVID-19 policy, school policy makers must define clear goals and select thresholds to add or remove mitigation measures based on these goals.

## Notes

Supplement. **eMethods 1.** Model Structure and Parameterization **eMethods 2.** Sources for Mitigation Ranges **eMethods 3.** Meta-Modeling Methods **eFigure 1.** Model-Estimated Mean Number of Additional Hospitalizations per 100 000 Individuals Over 30 Days in the Immediate School Community Associated With Reductions in Mitigation Effectiveness in the Simulated Elementary School Setting (With 70% Adult Vaccination, 70% Vaccine Effectiveness, and No Weekly Screening) **eFigure 2.** Sensitivity Analysis for 50% Adult Vaccination Rate (With Delta Variant, 70% Vaccine Effectiveness, and No Weekly Screening) **eFigure 3.** Sensitivity Analysis for Weekly Screening (With Delta Variant, 70% Adult Vaccination Rate, and 70% Vaccine Effectiveness) **eFigure 4.** Sensitivity Analysis for 50% Vaccine Effectiveness (With Delta Variant, 70% Adult Vaccination Rate, and No Weekly Screening) **eFigure 5.** Sensitivity Analysis for 25% Vaccine Effectiveness (With Delta Variant, 70% Adult Vaccination Rate, and No Weekly Screening) **eFigure 6.** Sensitivity Analysis for 90% Vaccine Effectiveness (With Delta Variant, 70% Adult Vaccination Rate, and No Weekly Screening) **eTable 1.** Observed Local Incidence Decision Thresholds (in Cases per 100 000 Residents per Day) for the Alpha Variant Baseline Scenario **eTable 2.** Observed Local Incidence Decision Thresholds (in Cases per 100 000 Residents per Day) for the Wild-Type Variant Baseline Scenario **eTable 3.** Observed Local Incidence Decision Thresholds (in Cases per 100 000 Residents per Day) for the 50% Adult Vaccination Rate Sensitivity Analysis **eTable 4.** Observed Local Incidence Decision Thresholds (in Cases per 100 000 Residents per Day) for the Weekly Screening Sensitivity Analysis **eTable 5.** Observed Local Incidence Decision Thresholds (in Cases per 100 000 Residents per Day) for the 50% Vaccine Effectiveness Sensitivity Analysis **eTable 6.** Observed Local Incidence Decision Thresholds (in Cases per 100 000 Residents per Day) for the 25% Vaccine Effectiveness Sensitivity Analysis **eTable 7.** Observed Local Incidence Decision Thresholds (in Cases per 100 000 Residents per Day) for the 90% Vaccine Effectiveness Sensitivity Analysis
eReferences.

## References

[1] US Centers for Disease Control and Prevention. Guidance for COVID-19 prevention in K-12 schools. Updated July 9, 2021. Accessed July 16, 2021. https://www.cdc.gov/coronavirus/2019-ncov/community/schools-childcare/k-12-guidance.html

[2] Balingit M, St. George D, Strauss V. As new school year looms, debates over mask mandates stir anger and confusion. *The Washington Post*. Published July 29, 2021. Accessed December 13, 2021. https://www.washingtonpost.com/education/2021/07/29/school-masks-coronavirus/

[3] Massachusetts Department of Elementary and Secondary Education. DESE policy on vaccination rate threshold. Published September 27, 2021. Accessed January 5, 2022. https://www.doe.mass.edu/covid19/on-desktop/2021-0927vax-rate-guidance.pdf

[4] Gewertz C. Some schools are dropping mask mandates. should yours? Education Week. Published November 3, 2021. Accessed December 13, 2021. https://www.edweek.org/leadership/some-schools-are-dropping-mask-mandates-should-yours/2021/11

[5] Brooks JT, Butler JC. Effectiveness of mask wearing to control community spread of SARS-CoV-2. JAMA. 2021;325(10):998-999. doi:10.1001/jama.2021.1505 33566056 PMC8892938

[6] IHME COVID-19 Forecasting Team. Modeling COVID-19 scenarios for the United States. Nat Med. 2021;27(1):94-105. doi:10.1038/s41591-020-1132-9 33097835 PMC7806509

[7] Abaluck J, Kwong LH, Styczynski A, . Impact of community masking on COVID-19: a cluster-randomized trial in Bangladesh. Science. 2021;eabi9069. doi:10.1126/science.abi9069 34855513 PMC9036942

[8] Chu DK, Akl EA, Duda S, Solo K, Yaacoub S, Schünemann HJ; COVID-19 Systematic Urgent Review Group Effort (SURGE) study authors. Physical distancing, face masks, and eye protection to prevent person-to-person transmission of SARS-CoV-2 and COVID-19: a systematic review and meta-analysis. Lancet. 2020;395(10242):1973-1987. doi:10.1016/S0140-6736(20)31142-9 32497510 PMC7263814

[9] US Centers for Disease Control and Prevention. Science brief: community use of masks to control the spread of SARS-CoV-2. Updated December 6, 2021. Accessed December 13, 2021. https://www.cdc.gov/coronavirus/2019-ncov/science/science-briefs/masking-science-sars-cov2.html

[10] Clapp PW, Sickbert-Bennett EE, Samet JM, ; US Centers for Disease Control and Prevention Epicenters Program. Evaluation of cloth masks and modified procedure masks as personal protective equipment for the public during the COVID-19 pandemic. JAMA Intern Med. 2021;181(4):463-469. doi:10.1001/jamainternmed.2020.8168 33300948 PMC7729588

[11] Doron S, Schechter-Perkins E, Branch-Elliman W. Some schools are requiring everyone to mask up—that doesn’t have to be permanent. *The Washington Post*. Published August 24, 2021. Accessed December 13, 2021. https://www.washingtonpost.com/outlook/2021/08/24/some-schools-are-requiring-everyone-mask-up-that-doesnt-have-be-permanent/

[12] Rowland LC, Klinkhammer MD, Ramirez DWE. Dynamic masking: a proposal of burden-based metrics for masking in K-12 schools during the COVID-19 pandemic. J Sch Health. 2021. doi:10.1111/josh.13099 34750833 PMC8661880

[13] American Academy of Pediatrics. COVID-19 guidance for safe schools and promotion of in-person learning. Updated November 11, 2021. Accessed December 13, 2021. https://www.aap.org/en/pages/2019-novel-coronavirus-covid-19-infections/clinical-guidance/covid-19-planning-considerations-return-to-in-person-education-in-schools/

[14] Grose J. We need to talk about an off-ramp for masking at school. *The New York Times*. Published October 29, 2021. Accessed December 13, 2021. https://www.nytimes.com/2021/10/29/opinion/mask-kids-vaccine.html

[15] Allen JG, Jenkins H. The hard COVID-19 questions we’re not asking. *The New York Times*. Published August 30, 2021. Accessed December 13, 2021. https://www.nytimes.com/2021/08/30/opinion/us-covid-policy.html

[16] Bilinski A, Salomon JA, Giardina J, Ciaranello A, Fitzpatrick MC. Passing the test: a model-based analysis of safe school-reopening strategies. Ann Intern Med. 2021;174(8):1090-1100. doi:10.7326/M21-0600 34097433 PMC8252151

[17] Husereau D, Drummond M, Petrou S, ; CHEERS Task Force. Consolidated Health Economic Evaluation Reporting Standards (CHEERS) statement. Value Health. 2013;16(2):e1-e5. doi:10.1016/j.jval.2013.02.010 23538200

[18] Lauer SA, Grantz KH, Bi Q, . The incubation period of coronavirus disease 2019 (COVID-19) from publicly reported confirmed cases: estimation and application. Ann Intern Med. 2020;172(9):577-582. doi:10.7326/M20-0504 32150748 PMC7081172

[19] He X, Lau EHY, Wu P, . Temporal dynamics in viral shedding and transmissibility of COVID-19. Nat Med. 2020;26(5):672-675. doi:10.1038/s41591-020-0869-5 32296168

[20] Li Q, Guan X, Wu P, . Early transmission dynamics in Wuhan, China, of novel coronavirus-infected pneumonia. N Engl J Med. 2020;382(13):1199-1207. doi:10.1056/NEJMoa2001316 31995857 PMC7121484

[21] Gatto M, Bertuzzo E, Mari L, . Spread and dynamics of the COVID-19 epidemic in Italy: effects of emergency containment measures. Proc Natl Acad Sci U S A. 2020;117(19):10484-10491. doi:10.1073/pnas.2004978117 32327608 PMC7229754

[22] Kerr CC, Stuart RM, Mistry D, Covasim: an agent-based model of COVID-19 dynamics and interventions. medRxiv. Preprint published April 1, 2021. doi:10.1101/2020.05.10.20097469 PMC8341708 34310589

[23] Firth JA, Hellewell J, Klepac P, . Combining fine-scale social contact data with epidemic modelling reveals interactions between contact tracing, quarantine, testing and physical distancing for controlling COVID-19. medRxiv. Preprint published July 2, 2020. doi:10.1101/2020.05.26.20113720

[24] Doyle T, Kendrick K, Troelstrup T, . COVID-19 in primary and secondary school settings during the first semester of school reopening—Florida, August-December 2020. MMWR Morb Mortal Wkly Rep. 2021;70(12):437-441. doi:10.15585/mmwr.mm7012e2 33764962 PMC7993553

[25] Davies NG, Abbott S, Barnard RC, ; CMMID COVID-19 Working Group; COVID-19 Genomics UK (COG-UK) Consortium. Estimated transmissibility and impact of SARS-CoV-2 lineage B.1.1.7 in England. Science. 2021;372(6538):eabg3055. doi:10.1126/science.abg3055 33658326 PMC8128288

[26] Singanayagam A, Hakki S, Dunning J, ; ATACCC Study Investigators. Community transmission and viral load kinetics of the SARS-CoV-2 Delta (B.1.617.2) variant in vaccinated and unvaccinated individuals in the UK: a prospective, longitudinal, cohort study. Lancet Infect Dis. 2021;S1473-3099(21)00648-4. doi:10.1016/S1473-3099(21)00648-4 34756186 PMC8554486

[27] Dougherty K, Mannell M, Naqvi O, Matson D, Stone J. SARS-CoV-2 B.1.617.2 (Delta) variant COVID-19 outbreak associated with a gymnastics facility—Oklahoma, April-May 2021. MMWR Morb Mortal Wkly Rep. 2021;70(28):1004-1007. doi:10.15585/mmwr.mm7028e2 34264910 PMC8314708

[28] National Centre for Immunisation Research and Surveillance. COVID-19 in schools and early childhood education and care services–the experience in NSW: 16 June to 31 July 2021. Accessed December 11, 2021. https://www.ncirs.org.au/sites/default/files/2021-09/NCIRS%20NSW%20Schools%20COVID_Summary_8%20September%2021_Final.pdf

[29] Thompson HA, Mousa A, Dighe A, . Severe acute respiratory syndrome coronavirus 2 (SARS-CoV-2) setting-specific transmission rates: a systematic review and meta-analysis. Clin Infect Dis. 2021;73(3):e754-e764. doi:10.1093/cid/ciab100 33560412 PMC7929012

[30] Byambasuren O, Cardona M, Bell K, Clark J, McLaws M-L, Glasziou P. Estimating the extent of asymptomatic COVID-19 and its potential for community transmission: systematic review and meta-analysis. J Assoc Med Microbiol Infect Dis Canada. 2020;5(4):223-234. doi:10.3138/jammi-2020-0030 PMC9602871 36340059

[31] He D, Zhao S, Lin Q, . The relative transmissibility of asymptomatic COVID-19 infections among close contacts. Int J Infect Dis. 2020;94:145-147. doi:10.1016/j.ijid.2020.04.034 32315808 PMC7166025

[32] Paul LA, Daneman N, Schwartz KL, . Association of age and pediatric household transmission of SARS-CoV-2 infection. JAMA Pediatr. 2021;175(11):1151-1158. doi:10.1001/jamapediatrics.2021.2770 34398179 PMC8369380

[33] Endo A, Abbott S, Kucharski AJ, Funk S; Centre for the Mathematical Modelling of Infectious Diseases COVID-19 Working Group. Estimating the overdispersion in COVID-19 transmission using outbreak sizes outside China. Wellcome Open Res. 2020;5:67. doi:10.12688/wellcomeopenres.15842.3 32685698 PMC7338915

[34] Fontanet A, Tondeur L, Grant R, . SARS-CoV-2 infection in schools in a northern French city: a retrospective serological cohort study in an area of high transmission, France, January to April 2020. Euro Surveill. 2021;26(15):2001695. doi:10.2807/1560-7917.ES.2021.26.15.2001695 33860747 PMC8167414

[35] Stein-Zamir C, Abramson N, Shoob H, . A large COVID-19 outbreak in a high school 10 days after schools’ reopening, Israel, May 2020. Euro Surveill. 2020;25(29). doi:10.2807/1560-7917.ES.2020.25.29.2001352 32720636 PMC7384285

[36] Han MS, Choi EH, Chang SH, . Clinical characteristics and viral RNA detection in children with coronavirus disease 2019 in the Republic of Korea. JAMA Pediatr. 2021;175(1):73-80. doi:10.1001/jamapediatrics.2020.3988 32857112 PMC7455883

[37] Atkeson A, Droste M, Mina MJ, Stock JH. Economic benefits of COVID-19 screening tests with a vaccine rollout. medRxiv. Preprint published March 5, 2021. doi:10.1101/2021.03.03.21252815

[38] Larremore DB, Wilder B, Lester E, . Test sensitivity is secondary to frequency and turnaround time for COVID-19 screening. Sci Adv. 2021;7(1):eabd5393. doi:10.1126/sciadv.abd5393 33219112 PMC7775777

[39] Cevik M, Tate M, Lloyd O, Maraolo AE, Schafers J, Ho A. SARS-CoV-2, SARS-CoV, and MERS-CoV viral load dynamics, duration of viral shedding, and infectiousness: a systematic review and meta-analysis. Lancet Microbe. 2021;2(1):e13-e22. doi:10.1016/S2666-5247(20)30172-5 33521734 PMC7837230

[40] Wyllie AL, Fournier J, Casanovas-Massana A, . Saliva or nasopharyngeal swab specimens for detection of SARS-CoV-2. N Engl J Med. 2020;383(13):1283-1286. doi:10.1056/NEJMc2016359 32857487 PMC7484747

[41] Kojima N, Turner F, Slepnev V, . Self-collected oral fluid and nasal swabs demonstrate comparable sensitivity to clinician collected nasopharyngeal swabs for coronavirus disease 2019 detection. Clin Infect Dis. 2021;73(9):e3106-e3109. doi:10.1093/cid/ciaa1589 33075138 PMC7665422

[42] US Centers for Disease Control and Prevention. COVID-19 pandemic planning scenarios. Updated March 19, 2021. Accessed July 27, 2021. https://www.cdc.gov/coronavirus/2019-ncov/hcp/planning-scenarios.html

[43] Delahoy MJ, Ujamaa D, Whitaker M, ; COVID-NET Surveillance Team; COVID-NET Surveillance Team. Hospitalizations associated with COVID-19 among children and adolescents—COVID-NET, 14 states, March 1, 2020-August 14, 2021. MMWR Morb Mortal Wkly Rep. 2021;70(36):1255-1260. doi:10.15585/mmwr.mm7036e2 34499627 PMC8437052

[44] Rosenberg ES, Dorabawila V, Easton D, COVID-19 vaccine effectiveness in New York State. N Engl J Med. Published online December 1, 2021. doi:10.1056/NEJMoa2116063 PMC8693697 34942067

[45] US Centers for Disease Control and Prevention. COVID-19 vaccinations in the United States. Updated December 12, 2021. Accessed December 13, 2021. https://covid.cdc.gov/covid-data-tracker/#vaccinations_vacc-total-admin-rate-total

[46] Rosenberg ES, Holtgrave DR, Dorabawila V, . New COVID-19 cases and hospitalizations among adults, by vaccination status—New York, May 3-July 25, 2021. MMWR Morb Mortal Wkly Rep. 2021;70(37):1306-1311. doi:10.15585/mmwr.mm7037a7 34529645 PMC8445378

[47] Keehner J, Horton LE, Binkin NJ, ; SEARCH Alliance. Resurgence of SARS-CoV-2 infection in a highly vaccinated health system workforce. N Engl J Med. 2021;385(14):1330-1332. doi:10.1056/NEJMc2112981 34469645 PMC8451183

[48] Fowlkes A, Gaglani M, Groover K, Thiese MS, Tyner H, Ellingson K; HEROES-RECOVER Cohorts. Effectiveness of COVID-19 vaccines in preventing SARS-CoV-2 infection among frontline workers before and during B.1.617.2 (Delta) variant predominance—eight U.S. locations, December 2020-August 2021. MMWR Morb Mortal Wkly Rep. 2021;70(34):1167-1169. doi:10.15585/mmwr.mm7034e4 34437521 PMC8389394

[49] Puranik A, Lenehan PJ, Silvert E, . Comparison of two highly-effective mRNA vaccines for COVID-19 during periods of Alpha and Delta variant prevalence. medRxiv. Preprint published August 9, 2021. doi:10.1101/2021.08.06.21261707

[50] Zeng B, Gao L, Zhou Q, Yu K, Sun F. Effectiveness of COVID-19 vaccines against SARS-CoV-2 variants of concern: a systematic review and meta-analysis. medRxiv. Preprint published September 26, 2021. doi:10.1101/2021.09.23.21264048 PMC9126103 35606843

[51] Vouriot CVM, Burridge HC, Noakes CJ, Linden PF. Seasonal variation in airborne infection risk in schools due to changes in ventilation inferred from monitored carbon dioxide. Indoor Air. 2021;31(4):1154-1163. doi:10.1111/ina.12818 33682974 PMC8251097

[52] Burridge HC, Bhagat RK, Stettler MEJ, The ventilation of buildings and other mitigating measures for COVID-19: a focus on wintertime. Proc Royal Soc A. 2021;477(2247):20200855. doi:10.1098/rspa.2020.0855 PMC8300604 35153550

[53] Rothamer DA, Sanders S, Reindl D, Bertram TH. Strategies to minimize SARS-CoV-2 transmission in classroom settings: combined impacts of ventilation and mask effective filtration efficiency. Sci Technol Built Environ. 2021;27(9):1181-1203. doi:10.1080/23744731.2021.1944665

[54] US Centers for Disease Control and Prevention. Science brief: transmission of SARS-CoV-2 in K-12 schools and early care and education programs—updated. Updated November 16, 2021. Accessed December 13, 2021. https://www.cdc.gov/coronavirus/2019-ncov/science/science-briefs/transmission_k_12_schools.html 34009772

[55] Falk A, Benda A, Falk P, Steffen S, Wallace Z, Høeg TB. COVID-19 cases and transmission in 17 K-12 schools—Wood County, Wisconsin, August 31-November 29, 2020. MMWR Morb Mortal Wkly Rep. 2021;70(4):136-140. doi:10.15585/mmwr.mm7004e3 33507890 PMC7842817

[56] Zimmerman KO, Brookhart MA, Kalu IC, Boutzoukas AE, McGann KA, Smith MJ, Maradiaga Panayotti GM, Armstrong SC, Weber DJ, Moorthy GS, Benjamin DK; ABC Science Collaborative. Community SARS-CoV-2 surge and within-school transmission. Pediatrics. 2021;148(4):e2021052686. doi:10.1542/peds.2021-052686 34321339 PMC10071552

[57] Jalal H, Dowd B, Sainfort F, Kuntz KM. Linear regression metamodeling as a tool to summarize and present simulation model results. Med Decis Making. 2013;33(7):880-890. doi:10.1177/0272989X13492014 23811758 PMC8008505

[58] R: A language and environment for statistical computing [computer program]. Version 4.0.2. Vienna, Austria: R Foundation for Statistical Computing; 2020.

[59] Bilinski A. BackToSchool2. Accessed January 5, 2022. https://github.com/abilinski/BackToSchool2

[60] Walter EB, Talaat KR, Sabharwal C, ; C4591007 Clinical Trial Group. Evaluation of the BNT162b2 COVID-19 vaccine in children 5 to 11 years of age. N Engl J Med. 2021. doi:10.1056/NEJMoa2116298 34752019 PMC8609605

[61] Bar-On YM, Goldberg Y, Mandel M, . Protection against COVID-19 by BNT162b2 booster across age groups. N Engl J Med. 2021. doi:10.1056/NEJMoa2114255 34879188 PMC8728796

[62] Andrews N, Stowe J, Kirsebom F, . Effectiveness of COVID-19 vaccines against the Omicron (B.1.1.529) variant of concern. Published December 12, 2021. Accessed January 5, 2022. https://khub.net/documents/135939561/430986542/Effectiveness+of+COVID-19+vaccines+against+Omicron+variant+of+concern.pdf/f423c9f4-91cb-0274-c8c5-70e8fad50074?t=1639154575915

[63] Ndugga N, Hill L, Artiga S, Haldar S. Latest data on COVID-19 vaccinations by race/ethnicity. Published December 2, 2021. Accessed December 13, 2021. https://www.kff.org/coronavirus-covid-19/issue-brief/latest-data-on-covid-19-vaccinations-by-race-ethnicity/

[64] Hamel L, Lopes L, Sparks G, . KFF COVID-19 vaccine monitor: October 2021. Published October 28, 2021. Accessed December 13, 2021. https://www.kff.org/coronavirus-covid-19/poll-finding/kff-covid-19-vaccine-monitor-october-2021/

[65] Tartof SY, Slezak JM, Fischer H, . Effectiveness of mRNA BNT162b2 COVID-19 vaccine up to 6 months in a large integrated health system in the USA: a retrospective cohort study. Lancet. 2021;398(10309):1407-1416. doi:10.1016/S0140-6736(21)02183-8 34619098 PMC8489881

