# Error in Figure 3

**DOI:** 10.1001/jamanetworkopen.2022.4819

**Published:** 2022-03-01

In the Original Investigation titled “Model-Estimated Association Between Simulated US Elementary School–Related SARS-CoV-2 Transmission, Mitigation Interventions, and Vaccine Coverage Across Local Incidence Levels,”^1^ published on February 14, 2022, there was an error in Figure 3. The footnote “a” should have only appeared once. This article has been corrected.^1^
