# Errors in Coding and Calculations of Hospital Rates

**DOI:** 10.1001/jamanetworkopen.2022.32401

**Published:** 2022-08-25

In the Original Investigation “Model-Estimated Association Between Simulated US Elementary School-Related SARS-CoV-2 Transmission, Mitigation Interventions, and Vaccine Coverage Across Local Incidence Levels,”^1^ published on February 14, 2022, a coding error resulted in an overestimate of the rate of additional hospitalizations associated with reductions in vaccine mitigation effectiveness. After correcting the error, the projected change in the hospitalization rate resulting from reductions in mitigation decreased, although the direction of the association between hospitalizations and mitigation effectiveness, vaccination coverage, and local incidence levels did not change. The authors have explained the errors and corrections in a Comment.^2^ In addition, there was an error in Figure 3; the maximum decision threshold reported in the main text from Figure 3 was associated with the 25% student vaccination coverage scenario, not the 50% coverage scenario. And there were 3 errors in the legends for eFigures 3 and 4 and eTable 6 in the Supplement. The corrections affect the previously reported decision thresholds in the Results and Discussion section of the main text, Figure 3, and eFigures 1 to 6 and eTables 1 to 7 in the Supplement. The errors and corrections do not affect results related to the 2 primary outcomes (probability of any in-school transmission and additional cases associated with reductions in mitigation effectiveness). The article has been corrected.^1^ There was a previous correction for a footnote error in Figure 3.^3^
