# Supplementary material for: Model-Estimated Association Between Simulated US Elementary School–Related SARS-CoV-2 Transmission, Mitigation Interventions, and Vaccine Coverage Across Local Incidence Levels
Source: JAMA Netw Open. 2022 Feb 14;5(2):e2147827. doi: 10.1001/jamanetworkopen.2021.47827 (PMC8845023; doi:10.1001/jamanetworkopen.2021.47827)
Supplement: Supplement. — eMethods 1. Model Structure and Parameterization eMethods 2. Sources for Mitigation Ranges eMethods 3. Meta-Modeling Methods eFigure 1. Model-Estimated Mean Number of Additional Hospitalizations per 100 000 Individuals Over 30 Days in the Immediate School Community Associated With Reductions in Mitigation Effectiveness in the Simulated Elementary School Setting (With 70% Adult Vaccination, 70% Vaccine Effectiveness, and No Weekly Screening) eFigure 2. Sensitivity Analysis for 50% Adult Vaccination Rate (With Delta Variant, 70% Vaccine Effectiveness, and No Weekly Screening) eFigure 3. Sensitivity Analysis for Weekly Screening (With Delta Variant, 70% Adult Vaccination Rate, and 70% Vaccine Effectiveness) eFigure 4. Sensitivity Analysis for 50% Vaccine Effectiveness (With Delta Variant, 70% Adult Vaccination Rate, and No Weekly Screening) eFigure 5. Sensitivity Analysis for 25% Vaccine Effectiveness (With Delta Variant, 70% Adult Vaccination Rate, and No Weekly Screening) eFigure 6. Sensitivity Analysis for 90% Vaccine Effectiveness (With Delta Variant, 70% Adult Vaccination Rate, and No Weekly Screening) eTable 1. Observed Local Incidence Decision Thresholds (in Cases per 100 000 Residents per Day) for the Alpha Variant Baseline Scenario eTable 2. Observed Local Incidence Decision Thresholds (in Cases per 100 000 Residents per Day) for the Wild-Type Variant Baseline Scenario eTable 3. Observed Local Incidence Decision Thresholds (in Cases per 100 000 Residents per Day) for the 50% Adult Vaccination Rate Sensitivity Analysis eTable 4. Observed Local Incidence Decision Thresholds (in Cases per 100 000 Residents per Day) for the Weekly Screening Sensitivity Analysis eTable 5. Observed Local Incidence Decision Thresholds (in Cases per 100 000 Residents per Day) for the 50% Vaccine Effectiveness Sensitivity Analysis eTable 6. Observed Local Incidence Decision Thresholds (in Cases per 100 000 Residents per Day) for the 25% Vaccine Effectiveness Sensitivity Analysis eTable [file jamanetwopen-e2147827-s001.pdf]

Supplemental Online Content

Giardina J, Bilinski A, Fitzpatrick MC, et al. Model-estimated association between simulated US elementary school–related SARS-CoV-2 transmission, mitigation interventions, and vaccine coverage across local incidence levels. *JAMA Netw Open.* 2022;5(2):e2147827.
doi:10.1001/jamanetworkopen.2021.47827

eMethods 1. Model Structure and Parameterization

eMethods 2. Sources for Mitigation Ranges

eMethods 3. Meta-Modeling Methods

eFigure 1. Model-Estimated Average Number of Additional Hospitalizations per 100 000 Individuals Over 30 Days in the Immediate School Community Associated With Reductions in Mitigation Effectiveness in the Simulated Elementary School Setting (With 70% Adult Vaccination, 70% Vaccine Effectiveness, and No Weekly Screening)

eFigure 2. Sensitivity Analysis for 50% Adult Vaccination Rate (With Delta Variant, 70% Vaccine Effectiveness, and No Weekly Screening)

eFigure 3. Sensitivity Analysis for Weekly Screening (With Delta Variant, 70% Adult Vaccination Rate, and 70% Vaccine Effectiveness)

eFigure 4. Sensitivity Analysis for 50% Vaccine Effectiveness (With Delta Variant, 70% Adult Vaccination Rate, and No Weekly Screening)

eFigure 5. Sensitivity Analysis for 25% Vaccine Effectiveness (With Delta Variant, 70% Adult Vaccination Rate, and No Weekly Screening)

eFigure 6. Sensitivity Analysis for 90% Vaccine Effectiveness (With Delta Variant, 70% Adult Vaccination Rate, and No Weekly Screening)

eTable 1. Observed Local Incidence Decision Thresholds (in Cases per 100 000 Residents per Day) for the Alpha Variant Baseline Scenario

eTable 2. Observed Local Incidence Decision Thresholds (in Cases per 100 000 Residents per Day) for the Wild-Type Variant Baseline Scenario

eTable 3. Observed Local Incidence Decision Thresholds (in Cases per 100 000 Residents per Day) for the 50% Adult Vaccination Rate Sensitivity Analysis

eTable 4. Observed Local Incidence Decision Thresholds (in Cases per 100 000 Residents per Day) for the Weekly Screening Sensitivity Analysis

eTable 5. Observed Local Incidence Decision Thresholds (in in Cases per 100 000 Residents per Day) for the 50% Vaccine Effectiveness Sensitivity Analysis

eTable 6. Observed Local Incidence Decision Thresholds (in Cases per 100 000 Residents per Day) for the 25% Vaccine Effectiveness Sensitivity Analysis

eTable 7. Observed Local Incidence Decision Thresholds (in Cases per 100 000 Residents per Day) for the 90% Vaccine Effectiveness Sensitivity Analysis

eReferences.

This supplemental material has been provided by the authors to give readers additional information about their work.

© 2022 Giardina J et al. *JAMA Network Open.*

eMethods 1. Model Structure and Parameterization

Basic Transmission Model and Course of Infection

The model used in this study, adapted from the model described by Bilinski, et al.[1], is an agent-based implementation of an SEIR model. Susceptible individuals who are exposed to an infectious individual have a daily probability of becoming infected determined by the daily attack rate, the location and length of the contact, the relative infectiousness of the infected contact (determined both by age and the potential for overdispersion among adults), and the relative susceptibility of the susceptible individual, as detailed in Table 1 in the main text – the probability of infection from a contact is the product of these terms. Following infection, an individual goes through an latent period determined by the distributions listed in Table 1. Following the latent period, an individual is infectious for a duration drawn from a lognormal distribution (as described in Table 1), and can either be asymptomatic or symptomatic, and clinical or subclinical (all asymptomatic individuals are subclinical, but not vice versa). Symptoms and clinical presentation of disease, if they appear, occur after infectiousness begins, with the length of time between exposure and presentation drawn from a gamma distribution (as described in Table 1). Following the infectious period, an individual cannot be reinfected.

Population

The simulated elementary school had 638 students across 6 grades, with 5 classes per grade. There is one teacher for each classroom and 30 additional staff members in the school, for a total of 60 teachers/staff. This synthetic population was developed by Bilinski, et al.[1], based on data from Wheaton[2] and National Center for Education Statistics[3], and is meant to reflect the average size of elementary schools in the US (weighted by the proportion of total elementary school students in the US attending each school) and the number and distribution of siblings within a school. Each student household includes two adults, with siblings assigned to the same household, and each teacher/staff household is assumed to have one additional adult.

Preventative Interventions

Vaccines

Individuals in the school community are randomly vaccinated according to the coverage rates in Table 1. Vaccinated individuals have a probability of being protected against infection equal to the vaccine efficacy; individuals are either fully protected or not protected at all through the course of the entire modeled period.

In-School Mitigation Interventions

The mitigation effectiveness described in the main text is applied as a multiplier to the full day in-school symptomatic adult-to-adult secondary attack rate listed in Table 1. For example, 40% mitigation effectiveness applied to the delta variant attack rate of 7% results in an actual attack rate of 4.2% (7%*(100% - 40%)). This mitigation is only applied to in-school contacts and is not applied to home or childcare contacts.

Isolation and Quarantine

Infected students and teachers/staff who present clinical symptoms are immediately isolated for 7 days and given a PCR test (with 100% sensitivity for symptomatic individuals). Once an individual has a positive result, all unvaccinated individuals in classrooms who had contact with that individual are quarantined for 7 days. (In order to reflect actual practices within schools, if an individual has a positive test result within three days of a classroom coming out of quarantine, that classroom is not re-quarantined.)

Weekly Screening

For the sensitivity analysis including weekly screening, all individuals at school (i.e., students and teachers/staff) are offered PCR tests every Monday. We assume that there is 90% uptake of the tests each week (randomly selected from the eligible population), and that the PCR tests have 90% sensitivity for asymptomatic individuals and a one-day turnaround time. Following the receipt of a positive test result, the infected individual and their classroom contacts are isolated and quarantined using the procedure described above.

Classroom and Schedule Structure

Students are randomly sorted into classrooms (within grades) and attend school 5 days a week (unless the student is isolated or classrooms are quarantined). In addition to interacting with students in their primary classroom, each day some classroom cohorts also attend "specials" classes (e.g., related arts) with a different teacher. On days when a student does not attend school (because it is a weekend), the student's household randomly interacts with another household with two parents present (from across the two households) to reflect shared childcare.

Seeded Infections

© 2022 Giardina J et al. *JAMA Network Open.*

The individuals in the school community are seeded daily with infections sourced from the wider local community. The daily probability that an individual not protected by a vaccine (see above) acquires an infection from the wider local community is equal to the actual local incidence rate (ie, the observed local incidence rate divided by the assumed percentage of cases that are observed) divided by the proportion of the population that is not protected by the vaccine (this normalization by the fraction of the susceptible population ensures that the rate of infection introductions into the school is equal to the actual local incidence rate). Since children currently make up a significant fraction of cases in the wider community,[4] children and adults were assumed to have equal local incidence rates. Also, note that it is assumed that this daily probability of infections sourced from the wider local community includes secondary infections from cases within a household that were directly sourced from the wider community. This means that, if a parent acquires a local community-sourced infection, the model does not allow them to pass it to their child (on the other hand, any school-sourced infections can be transmitted within a household). This assumption is made to ensure the rate of infections introduced to a school is not inflated beyond the actual local incidence rate, and that the model is focused on simulating the dynamics of school-related infections.

Secondary Attack Rates

We used previously reported findings to derive full day symptomatic adult-to-adult in-school secondary attack rates (SARs), defined as the proportion of susceptible adults exposed to a symptomatic adult index case who acquire SARS-CoV-2 infection per full school day of contact in the absence of mitigation, for wild-type virus, alpha variant, and delta variant. As in previous work, the adult-to-adult wild-type variant attack rate was 2%/day,[1] consistent with 2020-21 data from schools with minimal mitigation; a lower-bound estimate suggests a total in-school attack rate of 11%,[5] corresponding to a daily attack near 2% (assuming a constant daily attack rate over a 5-day infectious period). The transmissibility of the alpha variant is estimated at 59% higher than wild-type in the US,[6] so we assumed an attack rate of 3.5%/day.

To estimate the daily attack rate for the delta variant, we identified estimates for the overall household attack rates among unvaccinated populations since we apply the effects of vaccination within our model. The estimates for overall household attack rates included 38% in a UK-based study on symptomatic index cases,[7] 53% in a case study of an outbreak associated with an Oklahoma gymnastics facility,[8] and 71% in a government report from Australia on infections in homes where the index case was infected at school.[9] The estimate from the UK study was strictly for unvaccinated contacts, while the estimates from the Oklahoma and Australia studies were from populations with relatively low vaccination rates. We used the middle 53% estimate for the overall household attack in our model, which corresponds to a 14% daily household attack rate assuming a 5-day infectious period. Assuming that the daily household attack rate is double the in-school rate, to reflect a higher degree of contact within the home, and a 5-day infectious period, this estimate corresponds with about a 7%/day in-school attack rate.

This also aligns with a previous school modeling study, which assumed a twofold increase in transmissibility of delta compared to the alpha variant,[10] which would also suggest a 7%/day attack rate for delta compared to 3.5%/day for alpha. A case study of an elementary school outbreak estimated an overall classroom attack rate of about 50% from a single unvaccinated teacher,[11] which reflects a daily attack rate of about 13%, significantly higher than 7%/day. This is potentially a case of overdispersion in infectiousness, however, which is reflected in our model by applying a multiplier to individual infectiousness drawn from a lognormal distribution (see Table 1).[12,13] Within the model, we also apply reductions in the attack rate for asymptomatic adults (half as infectious),[14] children at school (half as infectious and half as susceptible),[1] and children at home (half as infectious if asymptomatic and half as susceptible).[1] We assumed symptomatic students were equally infectious as adults within the household setting to reflect recent evidence that younger children are potentially at least as infectious as older children in that setting, possibly because of increased contacts between younger children and their caregivers.[15]

Hospitalization risk

In order to parameterize the hospitalization risk among unvaccinated patients with SARS-CoV-2, we used estimates of the overall infection fatality rate (IFR) and the mortality rate among hospitalized patients, provided by the CDC for use in COVID-19 models.[16] If it is assumed that all patients who die are hospitalized prior to death, dividing the IFR by the mortality rate among hospitalized patients will recover the probability that an individual who has SARS-CoV-2 will be hospitalized (assuming, of course, that the IFR and hospitalized mortality rate are correctly estimated). The CDC parameter set provided estimates across four age groups: 0-17 years old, 18-49 years old, 50-64 years old, and 65+ years old. We used the parameters from the 18-49 years old age group for the unvaccinated adults in the model since parents of elementary school aged children are likely to fall into this age group, although some caregivers and teachers/staff could fall into higher age ranges. This age group had an IFR of 500/million and a 2.1% chance of death among hospitalized patients, leading to an estimated hospitalization risk of 2.4% among all unvaccinated adults.

The 0-17 years old age group had a 20/million IFR and 0.7% hospitalized mortality rate, resulting in an estimated 0.29% hospitalization risk for each infection in this age group. For the elementary student group, however, this estimate could not be directly applied in the model, since older children (e.g., 12-17 years old) in general have an observed increased risk of hospitalization.[17] Instead, given that preliminary seroprevalence estimates from the CDC show roughly equivalent cumulative incidence of SARS-CoV-2 infection between the 5-11 and 12-17 years old age groups,[18] and that a COVID-NET

study indicated cumulative incidence of hospitalization among the 5-11 year old group was about 38% of the cumulative incidence of the 12-17 year old group,[17] we multiplied the 0.29% hospitalization risk estimate by 38% to arrive at our final estimate of a 0.1% hospitalization risk of all unvaccinated students in the model.

Finally, we assumed that all vaccinated individuals had a negligible risk of hospitalization, since recent data has shown high vaccine effectiveness against hospitalization for ages <49.[19] Also, note that the 2% hospitalization rate used for adults is similar to inputs in other models, such as the low estimate used by Lemaitre, et al.[20] Still, this parameter should be considered to be highly uncertain and only used to provide a sense of the order of magnitude of hospitalizations resulting from cases in the immediate school community. The estimate of the number of hospitalizations from this parameter will probably be biased downward because (1) it does not consider wider community level effects of cases within the immediate school community (e.g., spread from students to elderly relatives) and (2) it assumes no hospitalization risk from breakthrough cases, which is not reflected in the available data, especially for older age groups – while vaccination greatly reduces the risk of hospitalization, it does not completely eliminate it.[19]

Vaccine Effectiveness

Although initial clinical trials in adults demonstrated vaccine efficacy >90%,[21,22] this likely wanes over time; observational data from the US during months when the delta variant predominated suggest vaccine effectiveness of 42-76%, in addition to a meta-analytic estimate of 72%.[23-27] In the base case, we modeled vaccine effectiveness of 70% in preventing all infections among students, educators/staff, and household members. We also conducted sensitivity analyses with 90%, 50%, and 25% effectiveness. The higher values (e.g., 90%) may more accurately reflect the impact of very recent vaccination for children (before waning vaccine effectiveness occurs)[28] and/or booster vaccinations for adults,[29] and the lower values (25%, 50%) may reflect values in the future, with further waning or new variants, including omicron.[30]

© 2022 Giardina J et al. *JAMA Network Open.*

eMethods 2. Sources for Mitigation Ranges

***A**: Simple ventilation and handwashing (open windows if present, portable air filters, maintain existing HVAC systems, and regular handwashing): 20-40% assumed effectiveness*

Vouriot, et al.[31] estimate that seasonal changes in ventilation increase secondary infections by 30-40% in fall and 80-90% in winter relative to summer, but note there is wide variation based on classroom activities. If an intervention replicates summer-levels of ventilation, this would correspond to a 23-29% reduction in the attack rate in the fall and a 44-47% reduction in the winter. Burridge, et al.[32] also present a wide range of studies on ventilation and surface cleaning, some of which show good ventilation (i.e., opening windows) could reduce the risk of infections by about half in an office setting (which is often less active than a classroom). Data from airflow studies estimate reduction in exposure to aerosols of 65% with portable HEPA filters.[33] Combining these data, we estimated a range of 20-40% risk reduction (since most classrooms will not have access to portable air cleaners).

***B**: Interventions in A, plus universal masking (a policy of masking all students and educators/staff): 60-80% assumed effectiveness*

There are few studies on the specific combination of masking and ventilation/handwashing that do not consider other interventions as well. Considering data from studies of masks alone, a meta-analysis estimated that using non-medical masks was, depending on the model used, associated with a 43% or 35% reduction in respiratory virus infection risk.[34] A recent study from Abaluck, et al.[35] found that a cluster randomized intervention in Bangladesh to promote the use of masks increased mask use by 29% and was associated with a 9.3% percentage point decrease in COVID-19 seroprevalence. The authors note that the effect of universal masking would likely be several times higher; they report a simple instrumental variable analysis in the supplementary material that estimates a 32% reduction in seroprevalence from universal masking, which is in line with the meta-analysis estimates on the reduction in infection risk from masking alone. Similarly, a meta-analysis on mask effectiveness across studies focusing on SARS-CoV-1, MERS, and SARS-CoV-2 by Chu, et al.[36] found a 44% reduction in risk for masks used in the community. There is substantial uncertainty in this estimate, with many studies reporting higher risk reductions.[37] Additionally, data from studies of simulated respiratory particles demonstrate fitted filtration efficiency values (proportion of particles kept behind a mask) of up to about 80% with consumer grade masks.[38,39] An experimental and mathematical modeling study on aerosol dynamics from Rothamer, et al.[40] estimated that a non-medical procedure mask with a baseline effectiveness of 29% had an effectiveness of 62-77% when combined with different levels of ventilation, with this combined effectiveness increasing for masks with better baseline effectiveness. This is also consistent with the meta-analysis from Chu, et al.[36] on masking in a healthcare setting (likely to be accompanied by ventilation; 70% reduction in risk) and a range of case studies suggesting masking effectiveness can reach as high as 70-79% in non-school settings, including in some households in China and during an outbreak on a US Navy ship.[37] We anticipated that the combined effectiveness of both masking and ventilation/handwashing (B) would be between the effectiveness estimates for mitigation groups A and C, and based on the data above, we assumed that masking and ventilation/handwashing was approximately 60-80% effective.

***C**: Combination interventions as implemented in many settings in the 2020-2021 school year (includes B, plus physical distancing of 3-6 feet when masked and >6 when unmasked, daily cleaning of surfaces, restrictions on shared items, and cohorting of students):[41] 90-100% assumed effectiveness*

This is the assumed maximum mitigation effect. CDC reports very effective in-school mitigation when the full package of interventions are implemented,[42] including those from Falk, et al.[43] and Zimmerman, et al.[44] Many studies reported total in-school secondary attack rates of 0.5-1.0% with implementation of this package of interventions; this corresponds to a 93% reduction on the unmitigated wild-type SAR.

© 2022 Giardina J et al. *JAMA Network Open.*

eMethods 3. Meta-Modeling Methods

The raw model output is highly stochastic and can only be evaluated at discrete parameter values, so to generate the presented smoothed heatmaps and associated contour lines, line graphs, and decision cutoffs across a continuous range of local incidence and mitigation effectiveness, we fit regressions for each outcome and associated scenario (e.g., at least one in-school transmission in the wild-type, 0% child vaccine, 70% adult vaccine scenario) as a function of observed local incidence and mitigation. This is essentially an application of regression meta-modeling, which is described by Jalal, et al.[45] as a way to summarize model results.

For each scenario (described in the main text), we ran 100 replicates of the base model for each combination of observed local incidence from 0 to 60 cases/100k/day (incremented by 1) and mitigation effectiveness from 0 to 100% (incremented by 1%) using the model code available at https://github.com/abilinski/BackToSchool2. (We only present 0 to 50 case notifications/100k/day in the study results to avoid boundary issues that can arise when fitting the regressions to stochastic output.)

For each outcome of interest (i.e., more than one in-school transmission, total number of cases, total number of hospitalizations), the dependent variable of the regression was the mean of 90 replicates in a training sample at each combination of incidence and mitigation. (Note that for the additional cases and hospitalization metrics, we fit the regression to the overall number of cases and hospitalizations in the immediate school community, and then subtracted the fitted regression across the different mitigation levels to generate the estimated average additional cases/hospitalizations from moving between each mitigation level.) We tested five specifications: linear, quadratic, cubic, and quartic polynomials, as well as linear regression with a log transformation on each predictor:

- Linear specification: $Outcome = \beta_0 + \beta_1 Incidence + \beta_2 Mitigation + \beta_3 Incidence * Mitigation$
- Quadratic specification: $Outcome = \sum_{k=0}^{2}\sum_{l=0}^{2}\beta_{k,l} Incidence^k * Mitigation^l$
- Cubic specification: $Outcome = \sum_{k=0}^{3}\sum_{l=0}^{3}\beta_{k,l} Incidence^k * Mitigation^l$
- Quartic specification: $Outcome = \sum_{k=0}^{4}\sum_{l=0}^{4}\beta_{k,l} Incidence^k * Mitigation^l$
- Log specification: $Outcome = \beta_0 + \beta_1 \ln Incidence + \beta_2 \ln Mitigation + \beta_3 \ln Incidence * \ln Mitigation$

For each combination of outcome measure and scenario, we selected the regression which minimized the root mean-squared prediction error in a hold-out test set containing 10 replicates (10%) at each combination of incidence and mitigation.

To assess how well the smoothing functions fit the expected value of the model output, we calculated the R^2 between binned averages of the model-generated outcomes in the hold-out test set and the average outcome predicted by the selected meta-model at the midpoint of the local incidence and mitigation values for each bin. We evaluated the fit for two different bin sizes: "large" bins, with a bin width of 5 for local incidence and 0.1 for mitigation effectiveness, and "small" bins with a bin width of 1 for local incidence and 0.1 for mitigation effectiveness. The lowest R^2 was 0.95 for the small bins and 0.98 for the large bins, indicating that the smoothing procedure to generate the figures accurately reflects the average model output within these bin sizes over the different scenarios analyzed.

eFigure 1. Model-Estimated Average Number of Additional Hospitalizations per 100 000 Individuals Over 30 Days in the Immediate School Community Associated With Reductions in Mitigation Effectiveness in the Simulated Elementary School Setting (With 70% Adult Vaccination, 70% Vaccine Effectiveness, and No Weekly Screening)

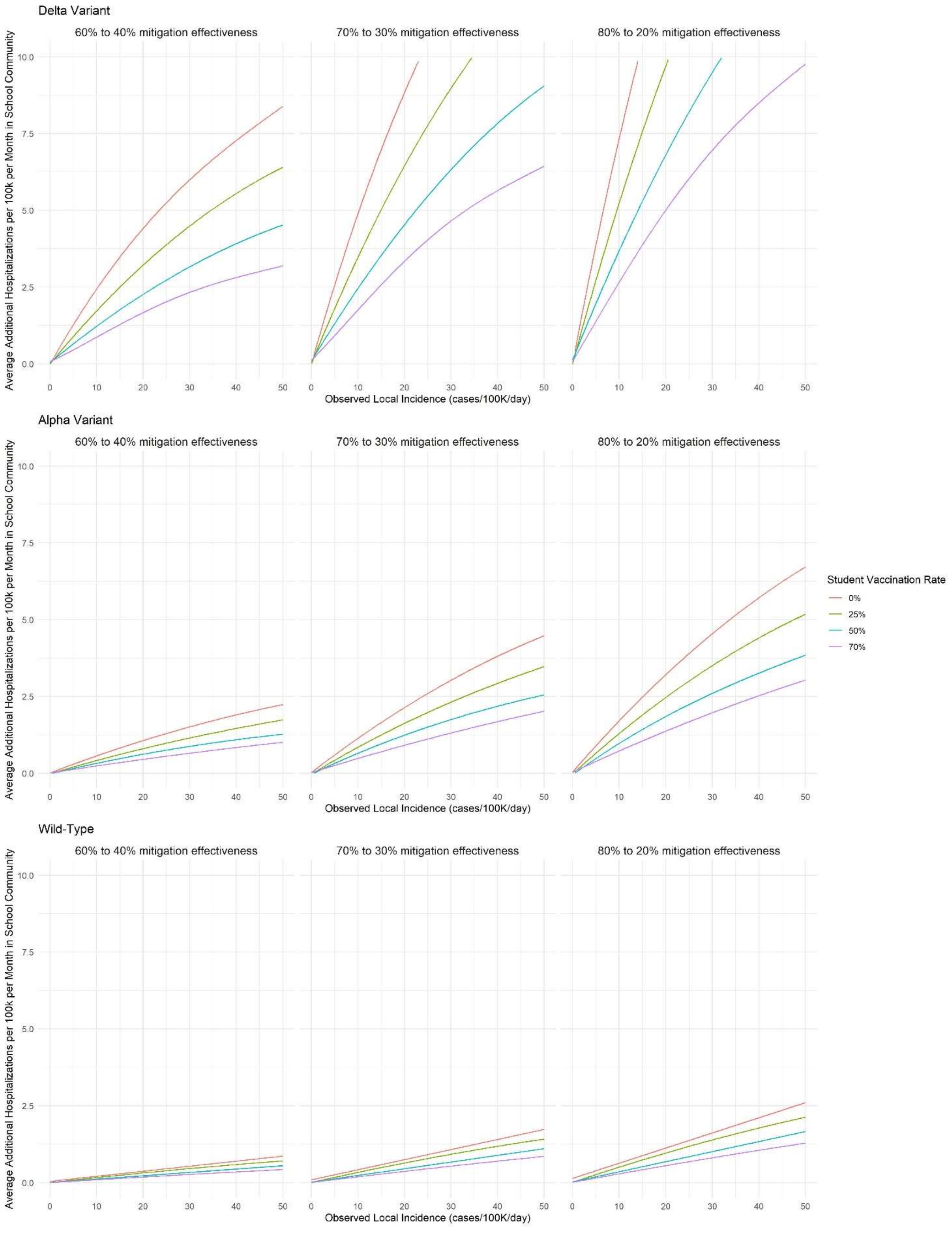

© 2022 Giardina J et al. *JAMA Network Open.*

eFigure 2. Sensitivity Analysis for 50% Adult Vaccination Rate (With Delta Variant, 70% Vaccine Effectiveness, and No Weekly Screening)

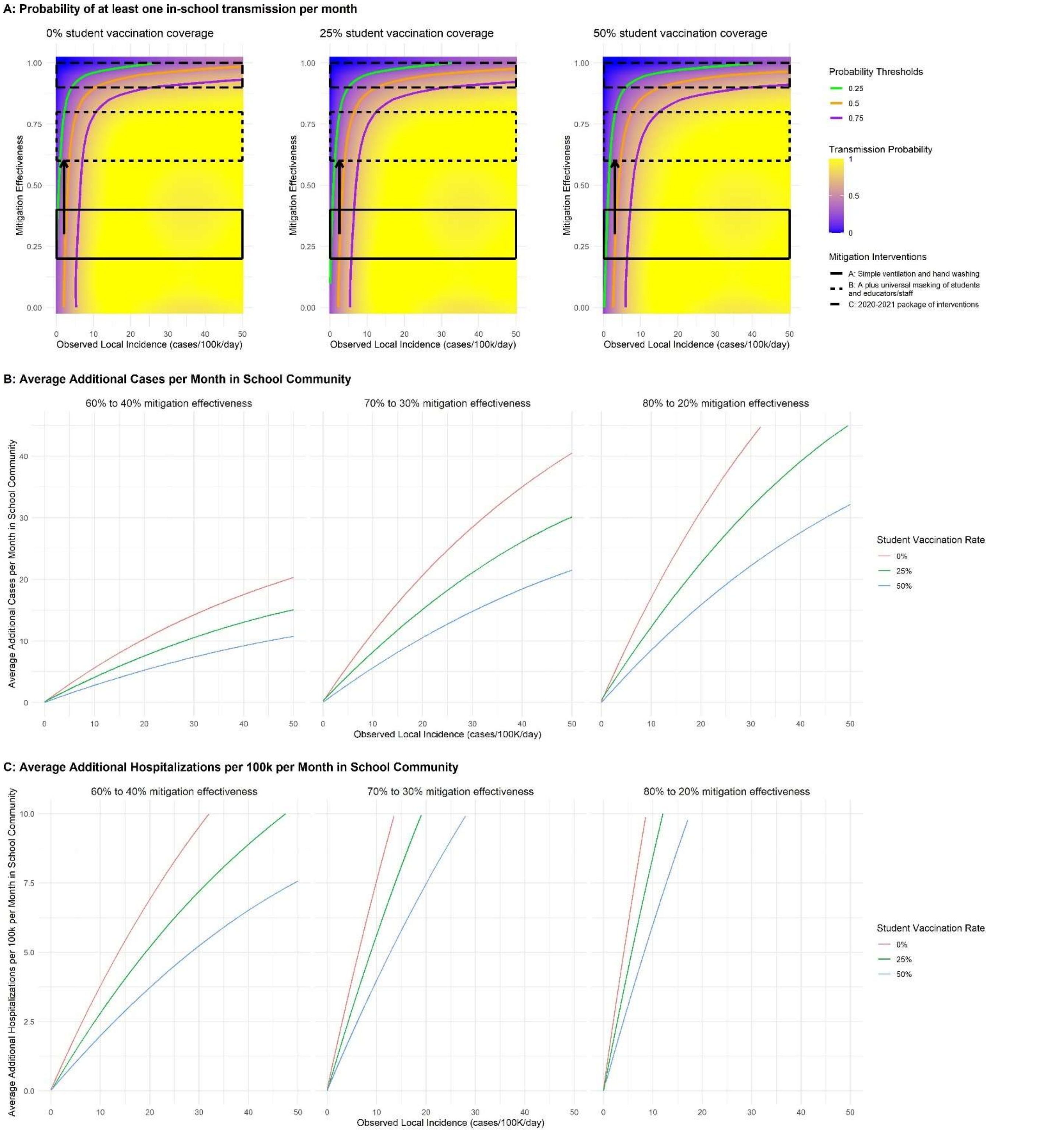

The row panels display the three outcomes analyzed in the study: (A) probability of at least one in-school transmission over 30 days; (B) model-estimated average number of additional cases over 30 days in the immediate school community associated with reductions in mitigation effectiveness; (C) model-estimated average number of additional hospitalizations per 100,000 individuals over 30 days in the immediate school community associated with reductions in mitigation effectiveness. The arrows in panel A indicate the local COVID-19 incidence rate at which a school might opt to move to the next more intensive mitigation strategy at a baseline 30% effectiveness, if the objective is to maintain the probability of at least one in-school transmission per month below 50%. 70% student vaccination coverage was not analyzed here because we assumed adult coverage would also be greater than student coverage.

© 2022 Giardina J et al. *JAMA Network Open.*

eFigure 3. Sensitivity Analysis for Weekly Screening (With Delta Variant, 70% Adult Vaccination Rate, and 70% Vaccine Effectiveness)

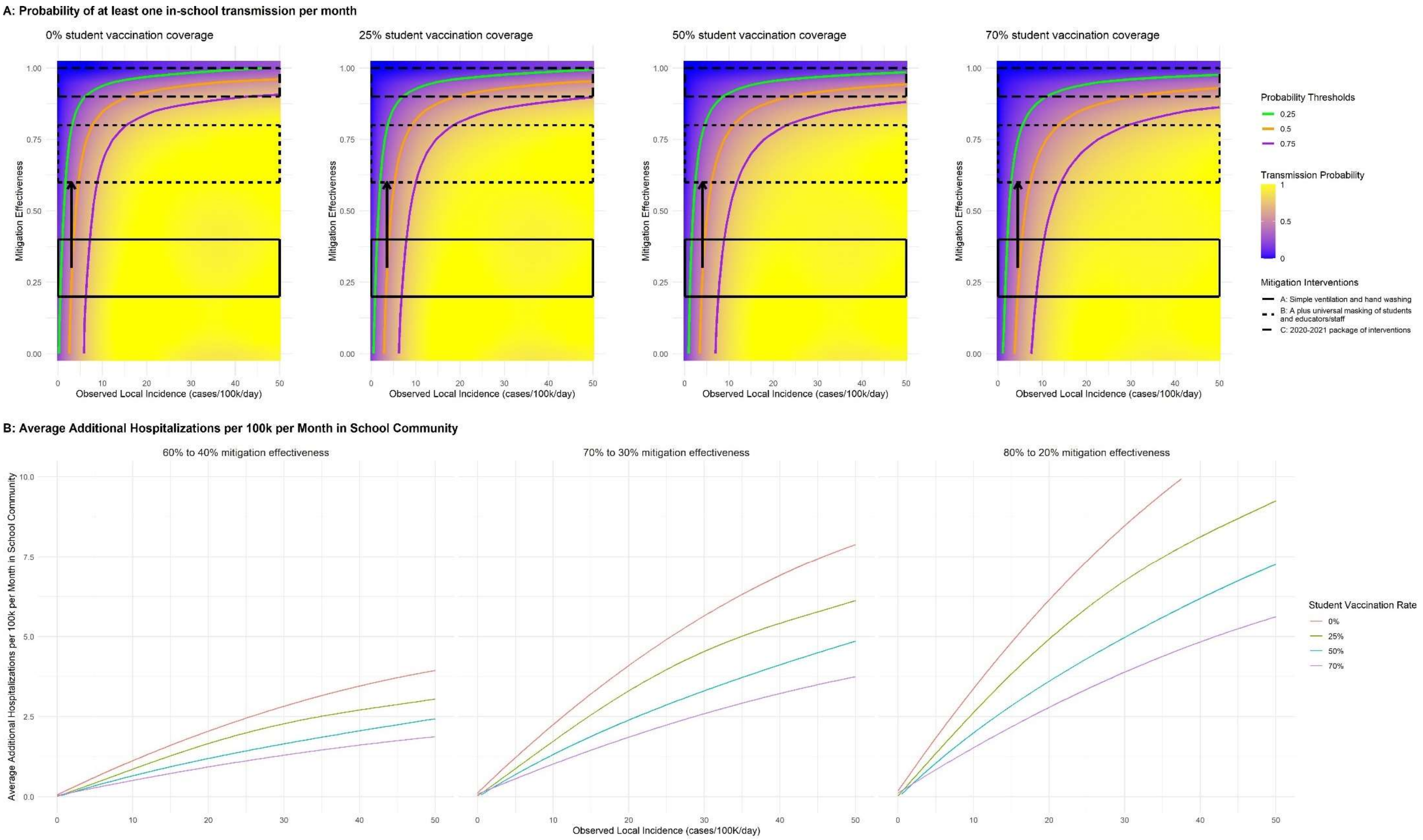

The row panels display two of the outcomes analyzed in the study: (A) probability of at least one in-school transmission over 30 days; (B) model-estimated average number of additional hospitalizations per 100,000 individuals over 30 days in the immediate school community associated with reductions in mitigation effectiveness. The arrows in panel A indicate the local COVID-19 incidence rate at which a school might opt to move to the next more intensive mitigation strategy at a baseline 30% effectiveness, if the objective is to maintain the probability of at least one in-school transmission per month below 50%. The third outcome (model-estimated average number of additional cases over 30 days in the immediate school community associated with reductions in mitigation effectiveness) is shown in Figure 3A, in the main text.

© 2022 Giardina J et al. *JAMA Network Open.*

eFigure 4. Sensitivity Analysis for 50% Vaccine Effectiveness (With Delta Variant, 70% Adult Vaccination Rate, and No Weekly Screening)

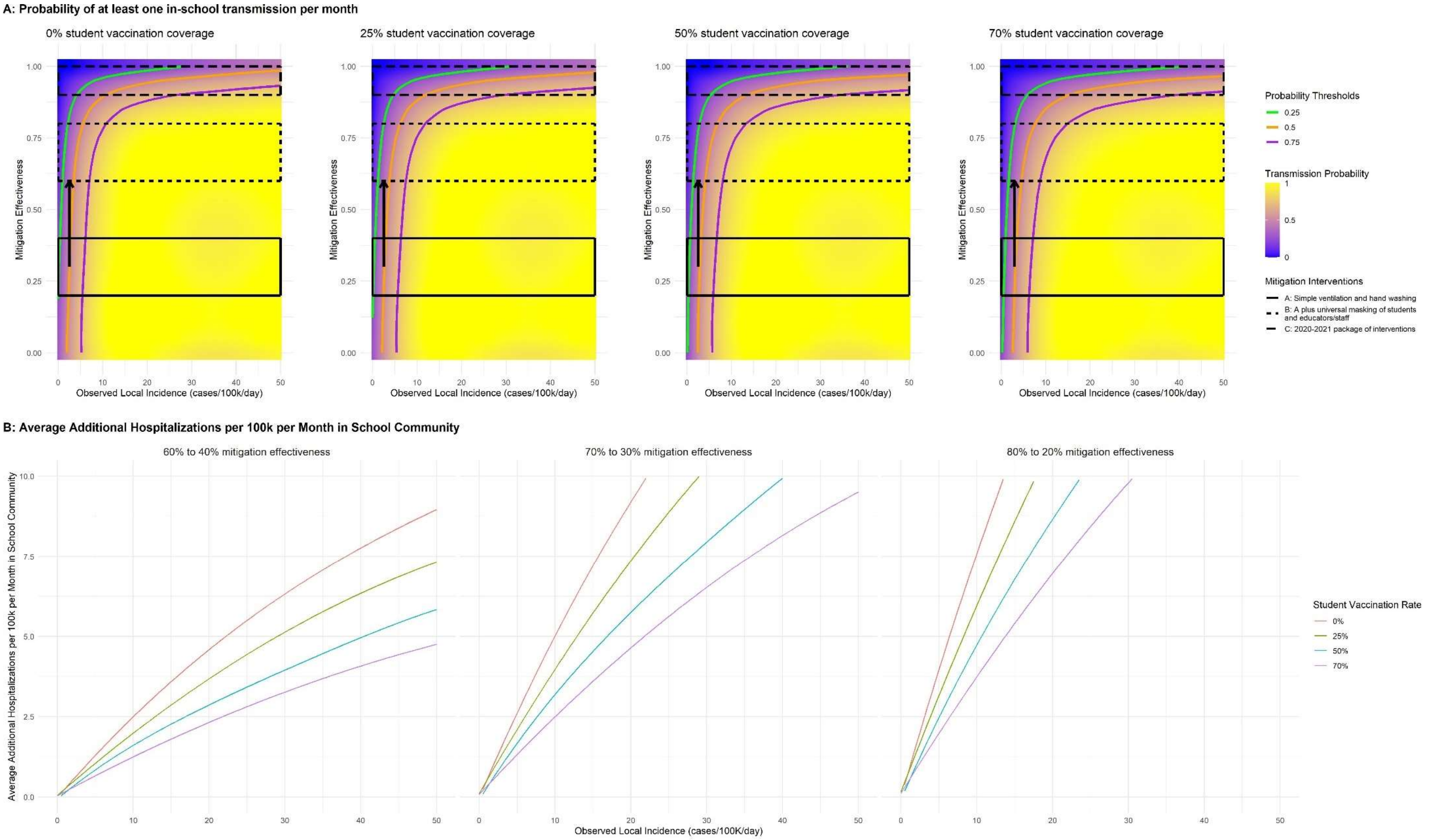

The row panels display two of the outcomes analyzed in the study: (A) probability of at least one in-school transmission over 30 days; (B) model-estimated average number of additional hospitalizations per 100,000 individuals over 30 days in the immediate school community associated with reductions in mitigation effectiveness. The arrows in panel A indicate the local COVID-19 incidence rate at which a school might opt to move to the next more intensive mitigation strategy at a baseline 30% effectiveness, if the objective is to maintain the probability of at least one in-school transmission per month below 50%. The third outcome (model-estimated average number of additional cases over 30 days in the immediate school community associated with reductions in mitigation effectiveness) is shown in Figure 3B, in the main text. Note that the hospitalization outcome assumes perfect vaccine protection against hospitalization, even with decreased protection against infection.

© 2022 Giardina J et al. *JAMA Network Open.*

eFigure 5. Sensitivity Analysis for 25% Vaccine Effectiveness (With Delta Variant, 70% Adult Vaccination Rate, and No Weekly Screening)

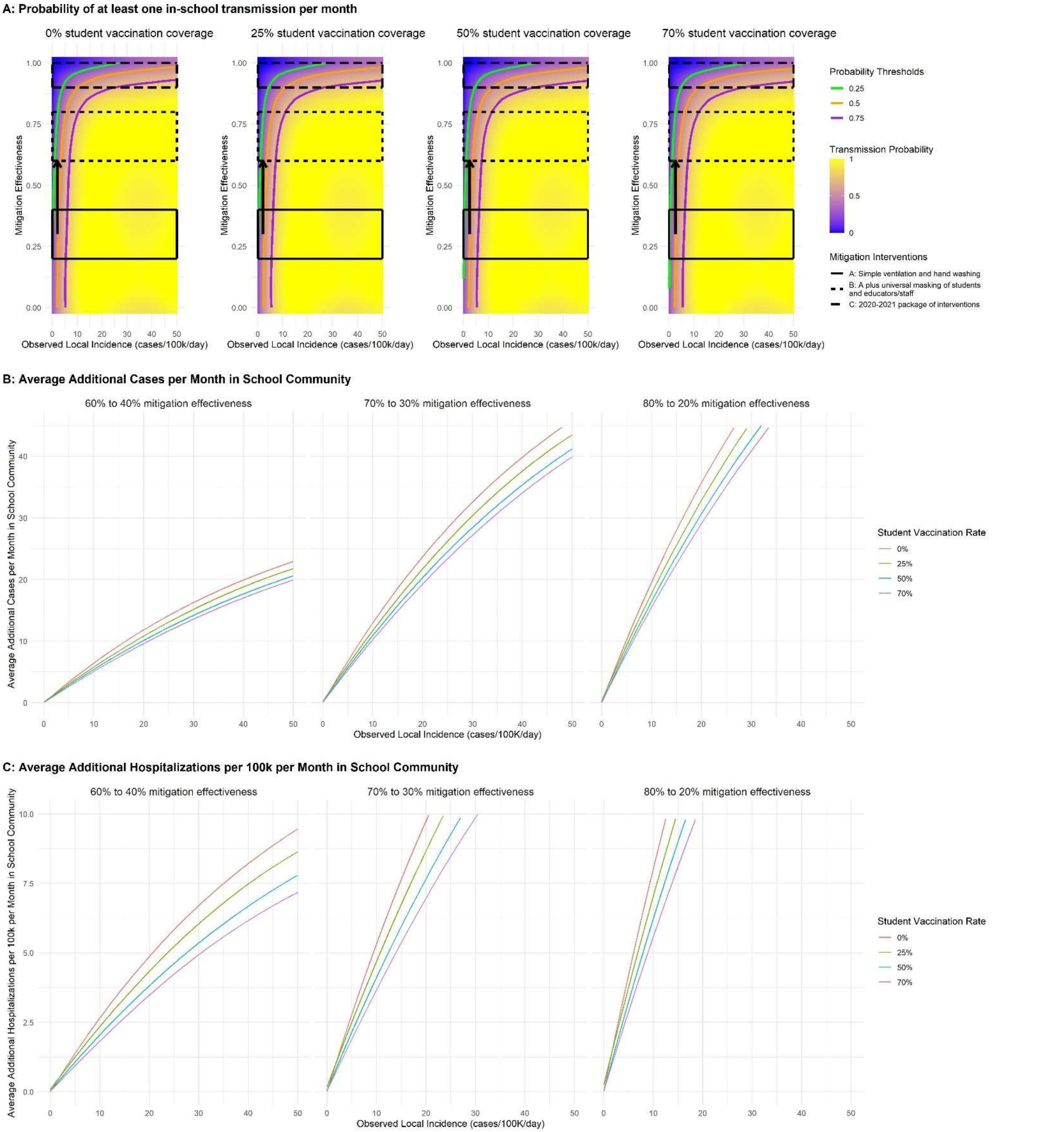

The row panels display the three outcomes analyzed in the study: (A) probability of at least one in-school transmission over 30 days; (B) model-estimated average number of additional cases over 30 days in the immediate school community associated with reductions in mitigation effectiveness; (C) model-estimated average number of additional hospitalizations per 100,000 individuals over 30 days in the immediate school community associated with reductions in mitigation effectiveness. The arrows in panel A indicate the local COVID-19 incidence rate at which a school might opt to move to the next more intensive mitigation strategy at a baseline 30% effectiveness, if the objective is to maintain the probability of at least one in-school transmission per month below 50%. Note that the hospitalization outcome assumes perfect vaccine protection against hospitalization, even with decreased protection against infection.

© 2022 Giardina J et al. *JAMA Network Open.*

eFigure 6. Sensitivity Analysis for 90% Vaccine Effectiveness (With Delta Variant, 70% Adult Vaccination Rate, and No Weekly Screening)

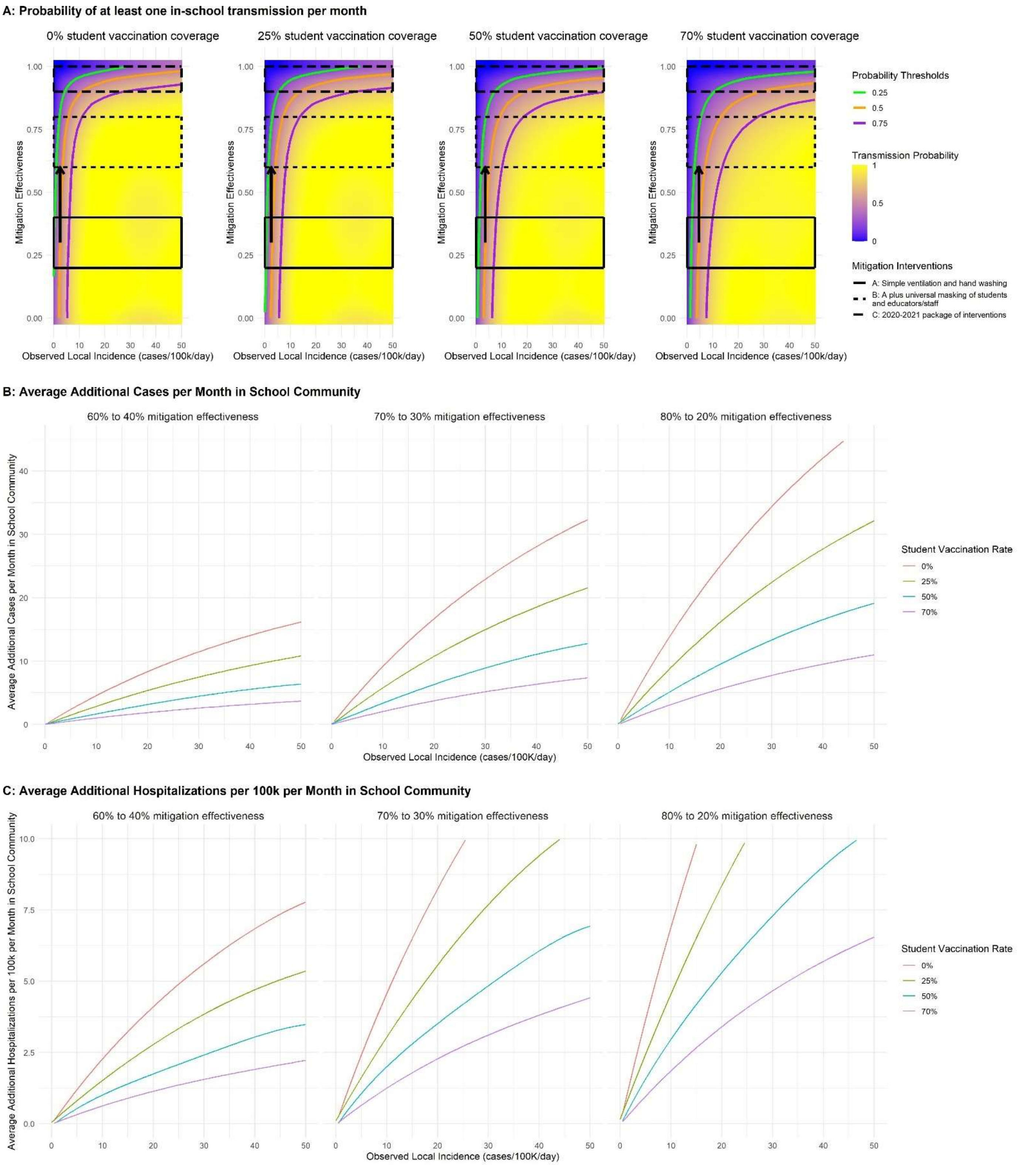

The row panels display the three outcomes analyzed in the study: (A) probability of at least one in-school transmission over 30 days; (B) model-estimated average number of additional cases over 30 days in the immediate school community associated with reductions in mitigation effectiveness; (C) model-estimated average number of additional hospitalizations per 100,000 individuals over 30 days in the immediate school community associated with reductions in mitigation effectiveness. The arrows in panel A indicate the local COVID-19 incidence rate at which a school might opt to move to the next more intensive mitigation strategy at a baseline 30% effectiveness, if the objective is to maintain the probability of at least one in-school transmission per month below 50%.

© 2022 Giardina J et al. *JAMA Network Open.*

eTable 1. Observed Local Incidence Decision Thresholds (in Cases per 100 000 Residents per Day) for the Alpha Variant Baseline Scenario

**Outcome**		Probability of at least 1 in-school transmission per month, with baseline mitigation effectiveness of:								
		40%			30%			20%		
**Decision Objective**		*To keep probability of at least 1 in-school transmission below:*								
		25%	50%	75%	25%	50%	75%	25%	50%	75%
		Baseline mitigation can only achieve objective at or below observed local incidence of:[a]								
**Student Vaccine Coverage**	0%	1	4	8	1	4	8	1	4	7
	25%	2	5	9	2	4	9	1	4	8
	50%	2	6	11	2	5	10	2	5	9
	70%	3	7	13	2	6	12	2	6	11
**Outcome**		Average additional cases per month associated with change in mitigation effectiveness:[b]								
		60% to 40%			70 to 30%			80% to 20%		
**Decision Objective**		*To keep average additional cases below:*								
		3 cases	5 cases	10 cases	3 cases	5 cases	10 cases	3 cases	5 cases	10 cases
		Mitigation can only be reduced at or below observed local incidence of:								
**Student Vaccine Coverage**	0%	23	42	>50	10	18	42	7	12	25
	25%	30	>50	>50	14	24	>50	9	15	34
	50%	44	>50	>50	19	35	>50	12	22	>50
	70%	>50	>50	>50	27	>50	>50	17	30	>50
**Outcome**		Average additional hospitalizations per 100,000 per month associated with change in mitigation effectiveness:[b]								
		60% to 40%			70 to 30%			80% to 20%		
**Decision Objective**		*To keep average additional hospitalizations per 100k below:*								
		1 hosp.	3 hosp.	5 hosp.	1 hosp.	3 hosp.	5 hosp.	1 hosp.	3 hosp.	5 hosp.
		Mitigation can only be reduced at or below observed local incidence of:								
**Student Vaccine Coverage**	0%	19	>50	>50	9	30	>50	6	19	34
	25%	25	>50	>50	12	41	>50	8	25	48
	50%	35	>50	>50	16	>50	>50	10	36	>50
	70%	49	>50	>50	22	>50	>50	14	49	>50

The alpha baseline scenario presented in this table reflects 70% adult vaccination coverage, 70% vaccine effectiveness, and no weekly screening. Units of observed local incidence thresholds are cases/100,000/day. It is assumed that 1/3 of all actual cases are observed.

[a]If observed local incidence is above these thresholds, additional mitigation measures beyond baseline will be needed to achieve each objective (e.g., keep probability of at least 1 in-school transmission per month below 50%).

[b]Only includes estimated average additional cases and hospitalizations in the immediate school community (students, teachers/staff, and household members). The potential for additional cases in the wider community stemming from in-school transmission was not modeled.

© 2022 Giardina J et al. *JAMA Network Open.*

eTable 2. Observed Local Incidence Decision Thresholds (in Cases per 100 000 Residents per Day) for the Wild-Type Variant Baseline Scenario

**Outcome**		Probability of at least 1 in-school transmission per month, with baseline mitigation effectiveness of:								
		40%			30%			20%		
**Decision Objective**		*To keep probability of at least 1 in-school transmission below:*								
		25%	50%	75%	25%	50%	75%	25%	50%	75%
		Baseline mitigation can only achieve objective at or below observed local incidence of:[a]								
**Student Vaccine Coverage**	0%	3	7	13	2	6	11	2	5	10
	25%	3	7	15	3	7	13	2	6	12
	50%	4	9	18	3	8	16	3	7	14
	70%	5	11	22	4	10	19	4	8	17
**Outcome**		Average additional cases per month associated with change in mitigation effectiveness:[b]								
		60% to 40%			70 to 30%			80% to 20%		
**Decision Objective**		*To keep additional cases below:*								
		3 cases	5 cases	10 cases	3 cases	5 cases	10 cases	3 cases	5 cases	10 cases
		Mitigation can only be reduced at or below observed local incidence of:								
**Student Vaccine Coverage**	0%	>50	>50	>50	26	48	>50	17	29	>50
	25%	>50	>50	>50	33	>50	>50	21	38	>50
	50%	>50	>50	>50	47	>50	>50	30	>50	>50
	70%	>50	>50	>50	>50	>50	>50	39	>50	>50
**Outcome**		Average additional hospitalizations per 100,000 per month associated with change in mitigation effectiveness:[b]								
		60% to 40%			70 to 30%			80% to 20%		
**Decision Objective**		*To keep additional hospitalizations per 100k below:*								
		1 hosp.	3 hosp.	5 hosp.	1 hosp.	3 hosp.	5 hosp.	1 hosp.	3 hosp.	5 hosp.
		Mitigation can only be reduced at or below observed local incidence of:								
**Student Vaccine Coverage**	0%	>50	>50	>50	28	>50	>50	17	>50	>50
	25%	>50	>50	>50	33	>50	>50	21	>50	>50
	50%	>50	>50	>50	45	>50	>50	30	>50	>50
	70%	>50	>50	>50	>50	>50	>50	38	>50	>50

The wild-type baseline scenario presented in this table reflects 70% adult vaccination coverage, 70% vaccine effectiveness, and no weekly screening. Units of observed local incidence thresholds are cases/100,000/day. It is assumed that 1/3 of all actual cases are observed.

[a]If observed local incidence is above these thresholds, additional mitigation measures beyond baseline will be needed to achieve each objective (e.g., keep probability of at least 1 in-school transmission per month below 50%).

[b]Only includes estimated average additional cases and hospitalizations in the immediate school community (students, teachers/staff, and household members). The potential for additional cases in the wider community stemming from in-school transmission was not modeled.

© 2022 Giardina J et al. *JAMA Network Open.*

eTable 3. Observed Local Incidence Decision Thresholds (in Cases per 100 000 Residents per Day) for the 50% Adult Vaccination Rate Sensitivity Analysis

**Outcome**		Probability of at least 1 in-school transmission per month, with baseline mitigation effectiveness of:								
		40%			30%			20%		
**Decision Objective**		*To keep probability of at least 1 in-school transmission below:*								
		25%	50%	75%	25%	50%	75%	25%	50%	75%
		Baseline mitigation can only achieve objective at or below observed local incidence of:[a]								
**Student Vaccine Coverage**	**0%**	<1	3	6	<1	2	6	<1	2	5
	25%	1	3	6	<1	3	6	<1	2	6
	50%	1	4	7	1	3	7	1	3	6
**Outcome**		Average additional cases per month associated with change in mitigation effectiveness:[b]								
		60% to 40%			70 to 30%			80% to 20%		
**Decision Objective**		*To keep average additional cases below:*								
		3 cases	5 cases	10 cases	3 cases	5 cases	10 cases	3 cases	5 cases	10 cases
		Mitigation can only be reduced at or below observed local incidence of:								
**Student Vaccine Coverage**	0%	5	9	19	2	4	9	2	3	6
	25%	7	12	28	3	6	12	2	4	8
	50%	11	19	45	5	9	19	3	6	12
**Outcome**		Average additional hospitalizations per 100,000 per month associated with change in mitigation effectiveness:[b]								
		60% to 40%			70 to 30%			80% to 20%		
**Decision Objective**		*To keep average additional hospitalizations per 100k below:*								
		1 hosp.	3 hosp.	5 hosp.	1 hosp.	3 hosp.	5 hosp.	1 hosp.	3 hosp.	5 hosp.
		Mitigation can only be reduced at or below observed local incidence of:								
**Student Vaccine Coverage**	0%	2	8	14	1	4	6	1	2	4
	25%	3	11	19	2	5	9	1	3	6
	50%	5	16	28	2	7	13	2	5	8

The sensitivity analysis presented in this table reflects the delta variant, 50% adult vaccination coverage, 70% vaccine effectiveness, and no weekly screening. 70% student vaccination coverage was not analyzed here because we assumed adult coverage would also be greater than student coverage. Units of observed local incidence thresholds are cases/100,000/day. It is assumed that 1/3 of all actual cases are observed.

[a]If observed local incidence is above these thresholds, additional mitigation measures beyond baseline will be needed to achieve each objective (e.g., keep probability of at least 1 in-school transmission per month below 50%).

[b]Only includes estimated average additional cases and hospitalizations in the immediate school community (students, teachers/staff, and household members). The potential for additional cases in the wider community stemming from in-school transmission was not modeled.

© 2022 Giardina J et al. *JAMA Network Open.*

eTable 4. Observed Local Incidence Decision Thresholds (in Cases per 100 000 Residents per Day) for the Weekly Screening Sensitivity Analysis

**Outcome**		Probability of at least 1 in-school transmission per month, with baseline mitigation effectiveness of:								
		40%			30%			20%		
**Decision Objective**		*To keep probability of at least 1 in-school transmission below:*								
		25%	50%	75%	25%	50%	75%	25%	50%	75%
		Baseline mitigation can only achieve objective at or below observed local incidence of:[a]								
**Student Vaccine Coverage**	0%	1	4	7	1	3	7	<1	3	6
	25%	1	4	8	1	4	7	1	3	7
	50%	2	5	9	1	4	8	1	4	8
	70%	2	5	10	2	5	9	2	4	9
**Outcome**		Average additional cases per month associated with change in mitigation effectiveness:[b]								
		60% to 40%			70 to 30%			80% to 20%		
**Decision Objective**		*To keep average additional cases below:*								
		3 cases	5 cases	10 cases	3 cases	5 cases	10 cases	3 cases	5 cases	10 cases
		Mitigation can only be reduced at or below observed local incidence of:								
**Student Vaccine Coverage**	0%	13	24	>50	6	11	24	4	7	15
	25%	18	34	>50	8	15	34	5	9	20
	50%	26	>50	>50	11	21	>50	7	13	29
	70%	36	>50	>50	16	29	>50	10	18	41
**Outcome**		Average additional hospitalizations per 100,000 per month associated with change in mitigation effectiveness:[b]								
		60% to 40%			70 to 30%			80% to 20%		
**Decision Objective**		*To keep average additional hospitalizations per 100k below:*								
		1 hosp.	3 hosp.	5 hosp.	1 hosp.	3 hosp.	5 hosp.	1 hosp.	3 hosp.	5 hosp.
		Mitigation can only be reduced at or below observed local incidence of:								
**Student Vaccine Coverage**	0%	9	33	>50	4	14	26	2	9	16
	25%	12	49	>50	6	18	35	4	12	20
	50%	16	>50	>50	7	26	>50	5	16	30
	70%	22	>50	>50	10	36	>50	6	22	42

The sensitivity analysis presented in this table reflects the delta variant, 70% adult vaccination coverage, 70% vaccine effectiveness, and weekly screening (90% uptake). Units of observed local incidence thresholds are cases/100,000/day. It is assumed that 1/3 of all actual cases are observed.

[a]If observed local incidence is above these thresholds, additional mitigation measures beyond baseline will be needed to achieve each objective (e.g., keep probability of at least 1 in-school transmission per month below 50%).

[b]Only includes estimated average additional cases and hospitalizations in the immediate school community (students, teachers/staff, and household members). The potential for additional cases in the wider community stemming from in-school transmission was not modeled.

© 2022 Giardina J et al. *JAMA Network Open.*

eTable 5. Observed Local Incidence Decision Thresholds (in in Cases per 100 000 Residents per Day) for the 50% Vaccine Effectiveness Sensitivity Analysis

**Outcome**		Probability of at least 1 in-school transmission per month, with baseline mitigation effectiveness of:								
		40%			30%			20%		
**Decision Objective**		*To keep probability of at least 1 in-school transmission below:*								
		25%	50%	75%	25%	50%	75%	25%	50%	75%
		Baseline mitigation can only achieve objective at or below observed local incidence of:[a]								
**Student Vaccine Coverage**	0%	<1	3	6	<1	3	6	<1	2	5
	25%	1	3	6	<1	3	6	<1	2	6
	50%	1	3	7	<1	3	6	<1	3	6
	70%	1	4	7	1	3	7	1	3	6
**Outcome**		Average additional cases per month associated with change in mitigation effectiveness:[b]								
		60% to 40%			70 to 30%			80% to 20%		
**Decision Objective**		*To keep average additional cases below:*								
		3 cases	5 cases	10 cases	3 cases	5 cases	10 cases	3 cases	5 cases	10 cases
		Mitigation can only be reduced at or below observed local incidence of:								
**Student Vaccine Coverage**	0%	5	9	19	3	4	9	2	3	6
	25%	6	11	25	3	5	11	2	3	7
	50%	8	14	33	4	7	14	3	4	9
	70%	10	18	42	5	9	18	3	5	11
**Outcome**		Average additional hospitalizations per 100,000 per month associated with change in mitigation effectiveness:[b]								
		60% to 40%			70 to 30%			80% to 20%		
**Decision Objective**		*To keep average additional hospitalizations per 100k below:*								
		1 hosp.	3 hosp.	5 hosp.	1 hosp.	3 hosp.	5 hosp.	1 hosp.	3 hosp.	5 hosp.
		Mitigation can only be reduced at or below observed local incidence of:								
**Student Vaccine Coverage**	0%	4	12	22	2	6	10	1	4	6
	25%	5	16	29	2	7	13	1	5	8
	50%	6	21	40	3	9	17	2	6	11
	70%	8	27	>50	4	12	22	2	8	14

The sensitivity analysis presented in this table reflects the delta variant, 70% adult vaccination coverage, 50% vaccine effectiveness, and no weekly screening. Note that the hospitalization outcome assumes perfect vaccine protection against hospitalization, even with decreased protection against infection. Units of observed local incidence thresholds are cases/100,000/day. It is assumed that 1/3 of all actual cases are observed.

[a]If observed local incidence is above these thresholds, additional mitigation measures beyond baseline will be needed to achieve each objective (e.g., keep probability of at least 1 in-school transmission per month below 50%).

[b]Only includes estimated average additional cases and hospitalizations in the immediate school community (students, teachers/staff, and household members). The potential for additional cases in the wider community stemming from in-school transmission was not modeled.

© 2022 Giardina J et al. *JAMA Network Open.*

eTable 6. Observed Local Incidence Decision Thresholds (in Cases per 100 000 Residents per Day) for the 25% Vaccine Effectiveness Sensitivity Analysis

**Outcome**		Probability of at least 1 in-school transmission per month, with baseline mitigation effectiveness of:								
		40%			30%			20%		
**Decision Objective**		*To keep probability of at least 1 in-school transmission below:*								
		25%	50%	75%	25%	50%	75%	25%	50%	75%
		Baseline mitigation can only achieve objective at or below observed local incidence of:[a]								
**Student Vaccine Coverage**	0%	<1	3	6	<1	2	6	<1	2	5
	25%	<1	3	6	<1	2	6	<1	2	5
	50%	<1	3	6	<1	3	6	<1	2	6
	70%	1	3	6	<1	3	6	<1	2	6
**Outcome**		Average additional cases per month associated with change in mitigation effectiveness:[b]								
		60% to 40%			70 to 30%			80% to 20%		
**Decision Objective**		*To keep average additional cases below:*								
		3 cases	5 cases	10 cases	3 cases	5 cases	10 cases	3 cases	5 cases	10 cases
		Mitigation can only be reduced at or below observed local incidence of:								
**Student Vaccine Coverage**	0%	4	8	16	2	4	8	1	2	5
	25%	5	9	18	2	4	8	1	3	5
	50%	5	9	20	3	4	9	2	3	6
	70%	6	10	21	3	5	10	2	3	6
**Outcome**		Average additional hospitalizations per 100,000 per month associated with change in mitigation effectiveness:[b]								
		60% to 40%			70 to 30%			80% to 20%		
**Decision Objective**		*To keep average additional hospitalizations per 100k below:*								
		1 hosp.	3 hosp.	5 hosp.	1 hosp.	3 hosp.	5 hosp.	1 hosp.	3 hosp.	5 hosp.
		Mitigation can only be reduced at or below observed local incidence of:								
**Student Vaccine Coverage**	0%	4	11	21	2	5	9	1	4	6
	25%	4	13	24	2	6	11	1	4	7
	50%	5	15	28	2	7	12	1	5	8
	70%	5	17	30	3	8	14	2	5	9

The sensitivity analysis presented in this table reflects the delta variant, 70% adult vaccination coverage, 50% vaccine effectiveness, and no weekly screening. Note that the hospitalization outcome assumes perfect vaccine protection against hospitalization, even with decreased protection against infection. Units of observed local incidence thresholds are cases/100,000/day. It is assumed that 1/3 of all actual cases are observed.

[a]If observed local incidence is above these thresholds, additional mitigation measures beyond baseline will be needed to achieve each objective (e.g., keep probability of at least 1 in-school transmission per month below 50%).

[b]Only includes estimated average additional cases and hospitalizations in the immediate school community (students, teachers/staff, and household members). The potential for additional cases in the wider community stemming from in-school transmission was not modeled.

© 2022 Giardina J et al. *JAMA Network Open.*

eTable 7. Observed Local Incidence Decision Thresholds (in Cases per 100 000 Residents per Day) for the 90% Vaccine Effectiveness Sensitivity Analysis

**Outcome**		Probability of at least 1 in-school transmission per month, with baseline mitigation effectiveness of:								
		40%			30%			20%		
**Decision Objective**		*To keep probability of at least 1 in-school transmission below:*								
		25%	50%	75%	25%	50%	75%	25%	50%	75%
		Baseline mitigation can only achieve objective at or below observed local incidence of:[a]								
**Student Vaccine Coverage**	0%	<1	3	6	<1	3	6	<1	2	5
	25%	1	3	7	1	3	6	<1	3	6
	50%	1	4	8	1	4	7	1	3	7
	70%	2	5	10	2	5	9	1	4	8
**Outcome**		Average additional cases per month associated with change in mitigation effectiveness:[b]								
		60% to 40%			70 to 30%			80% to 20%		
**Decision Objective**		*To keep average additional cases below:*								
		3 cases	5 cases	10 cases	3 cases	5 cases	10 cases	3 cases	5 cases	10 cases
		Mitigation can only be reduced at or below observed local incidence of:								
**Student Vaccine Coverage**	0%	6	11	25	3	5	11	2	3	7
	25%	10	18	44	5	9	18	3	6	12
	50%	19	35	>50	9	15	35	6	10	21
	70%	37	>50	>50	15	29	>50	10	17	43
**Outcome**		Average additional hospitalizations per 100,000 per month associated with change in mitigation effectiveness:[b]								
		60% to 40%			70 to 30%			80% to 20%		
**Decision Objective**		*To keep average additional hospitalizations per 100k below:*								
		1 hosp.	3 hosp.	5 hosp.	1 hosp.	3 hosp.	5 hosp.	1 hosp.	3 hosp.	5 hosp.
		Mitigation can only be reduced at or below observed local incidence of:								
**Student Vaccine Coverage**	0%	4	14	26	2	6	11	1	4	7
	25%	6	22	44	3	10	18	2	6	11
	50%	10	39	>50	5	16	31	3	10	19
	70%	17	>50	>50	8	29	>50	5	17	33

The sensitivity analysis presented in this table reflects the delta variant, 70% adult vaccination coverage, 90% vaccine effectiveness, and no weekly screening. Units of observed local incidence thresholds are cases/100,000/day. It is assumed that 1/3 of all actual cases are observed.

[a]If observed local incidence is above these thresholds, additional mitigation measures beyond baseline will be needed to achieve each objective (e.g., keep probability of at least 1 in-school transmission per month below 50%).

[b]Only includes estimated average additional cases and hospitalizations in the immediate school community (students, teachers/staff, and household members). The potential for additional cases in the wider community stemming from in-school transmission was not modeled.

© 2022 Giardina J et al. *JAMA Network Open.*

eReferences

1. Bilinski A, Salomon JA, Giardina J, Ciaranello A, Fitzpatrick MC. Passing the Test: A Model-Based Analysis of Safe School-Reopening Strategies. *Ann Intern Med.* 2021;174(8):1090-1100.
2. Wheaton WD. *U.S. Synthetic Population 2010 Version 1.0 Quick Start Guide.* RTI International; 2014.
3. National Center for Education Statistics. Number and percentage distribution of public elementary and secondary schools and enrollment, by level, type, and enrollment size of school: 2015-16, 2016-17, and 2017-18. *Digest of Education Statistics.* 2019.
4. Centers for Disease Control and Prevention. COVID-19 Weekly Cases and Deaths per 100,000 Population by Age, Race/Ethnicity, and Sex. https://covid.cdc.gov/covid-data-tracker/#demographicsovertime. Updated December 12, 2021. Accessed December 13, 2021.
5. Doyle T, Kendrick K, Troelstrup T, et al. COVID-19 in Primary and Secondary School Settings During the First Semester of School Reopening - Florida, August-December 2020. *MMWR Morb Mortal Wkly Rep.* 2021;70(12):437-441.
6. Davies NG, Abbott S, Barnard RC, et al. Estimated transmissibility and impact of SARS-CoV-2 lineage B.1.1.7 in England. *Science.* 2021;372(6538).
7. Singanayagam A, Hakki S, Dunning J, et al. Community transmission and viral load kinetics of the SARS-CoV-2 delta (B.1.617.2) variant in vaccinated and unvaccinated individuals in the UK: a prospective, longitudinal, cohort study. *Lancet Infect Dis.* 2021.
8. Dougherty K, Mannell M, Naqvi O, Matson D, Stone J. SARS-CoV-2 B.1.617.2 (Delta) Variant COVID-19 Outbreak Associated with a Gymnastics Facility - Oklahoma, April-May 2021. *MMWR Morb Mortal Wkly Rep.* 2021;70(28):1004-1007.
9. National Centre for Immunisation Research and Surveillance. COVID-19 in schools and early childhood education and care services – the experience in NSW: 16 June to 31 July 2021. https://www.ncirs.org.au/sites/default/files/2021-09/NCIRS%20NSW%20Schools%20COVID_Summary_8%20September%2021_Final.pdf. Published 2021. Accessed December 11, 2021.
10. Head JR, Andrejko KL, Remais JV. Model-based assessment of SARS-CoV-2 Delta variant transmission dynamics within partially vaccinated K-12 school populations. *Lancet Reg Health Am.* 2021:100133.
11. Lam-Hine T, McCurdy SA, Santora L, et al. Outbreak Associated with SARS-CoV-2 B.1.617.2 (Delta) Variant in an Elementary School - Marin County, California, May-June 2021. *MMWR Morb Mortal Wkly Rep.* 2021;70(35):1214-1219.
12. Kerr CC, Stuart RM, Mistry D, et al. Covasim: an agent-based model of COVID-19 dynamics and interventions. *medRxiv.* 2020:2020.2005.2010.20097469.
13. Endo A, Centre for the Mathematical Modelling of Infectious Diseases C-WG, Abbott S, Kucharski AJ, Funk S. Estimating the overdispersion in COVID-19 transmission using outbreak sizes outside China. *Wellcome Open Res.* 2020;5:67.
14. Byambasuren O, Cardona M, Bell K, Clark J, McLaws M-L, Glasziou P. Estimating the extent of asymptomatic COVID-19 and its potential for community transmission: Systematic review and meta-analysis. *Journal of the Association of Medical Microbiology and Infectious Disease Canada.* 2020;5(4):223-234.
15. Paul LA, Daneman N, Schwartz KL, et al. Association of Age and Pediatric Household Transmission of SARS-CoV-2 Infection. *JAMA Pediatr.* 2021;175(11):1151-1158.
16. Centers for Disease Control and Prevention. COVID-19 Pandemic Planning Scenarios. https://www.cdc.gov/coronavirus/2019-ncov/hcp/planning-scenarios.html. Updated March 19, 2021. Accessed July 27, 2021.
17. Delahoy MJ, Ujamaa D, Whitaker M, et al. Hospitalizations Associated with COVID-19 Among Children and Adolescents - COVID-NET, 14 States, March 1, 2020-August 14, 2021. *MMWR Morb Mortal Wkly Rep.* 2021;70(36):1255-1260.
18. Jones J. Epidemiology of COVID-19 in Children Aged 5 – 11 years. Centers for Disease Control and Prevention. https://www.cdc.gov/vaccines/acip/meetings/downloads/slides-2021-11-2-3/03-COVID-Jefferson-508.pdf. Published November 2, 2021. Accessed December 11, 2021.
19. Rosenberg ES, Dorabawila V, Easton D, et al. Covid-19 Vaccine Effectiveness in New York State. *New England Journal of Medicine.* 2021.
20. Lemaitre JC, Grantz KH, Kaminsky J, et al. A scenario modeling pipeline for COVID-19 emergency planning. *Sci Rep.* 2021;11(1):7534.
21. Polack FP, Thomas SJ, Kitchin N, et al. Safety and Efficacy of the BNT162b2 mRNA Covid-19 Vaccine. *N Engl J Med.* 2020;383(27):2603-2615.
22. Baden LR, El Sahly HM, Essink B, et al. Efficacy and Safety of the mRNA-1273 SARS-CoV-2 Vaccine. *N Engl J Med.* 2021;384(5):403-416.

23. Rosenberg ES, Holtgrave DR, Dorabawila V, et al. New COVID-19 Cases and Hospitalizations Among Adults, by Vaccination Status - New York, May 3-July 25, 2021. *MMWR Morb Mortal Wkly Rep.* 2021;70(37):1306-1311.
24. Keehner J, Horton LE, Binkin NJ, et al. Resurgence of SARS-CoV-2 Infection in a Highly Vaccinated Health System Workforce. *N Engl J Med.* 2021;385(14):1330-1332.
25. Fowlkes A, Gaglani M, Groover K, et al. Effectiveness of COVID-19 Vaccines in Preventing SARS-CoV-2 Infection Among Frontline Workers Before and During B.1.617.2 (Delta) Variant Predominance - Eight U.S. Locations, December 2020-August 2021. *MMWR Morb Mortal Wkly Rep.* 2021;70(34):1167-1169.
26. Puranik A, Lenehan PJ, Silvert E, et al. Comparison of two highly-effective mRNA vaccines for COVID-19 during periods of Alpha and Delta variant prevalence. *medRxiv.* 2021.
27. Zeng B, Gao L, Zhou Q, Yu K, Sun F. Effectiveness of COVID-19 vaccines against SARS-CoV-2 variants of concern: a systematic review and meta-analysis. *medRxiv.* 2021:2021.2009.2023.21264048.
28. Walter EB, Talaat KR, Sabharwal C, et al. Evaluation of the BNT162b2 Covid-19 Vaccine in Children 5 to 11 Years of Age. *New England Journal of Medicine.* 2021.
29. Bar-On YM, Goldberg Y, Mandel M, et al. Protection against Covid-19 by BNT162b2 Booster across Age Groups. *New England Journal of Medicine.* 2021.
30. Andrews N, Stowe J, Kirsebom F, et al. Effectiveness of COVID-19 vaccines against the Omicron (B.1.1.529) variant of concern. https://khub.net/documents/135939561/430986542/Effectiveness+of+COVID-19+vaccines+against+Omicron+variant+of+concern.pdf/f423c9f4-91cb-0274-c8c5-70e8fad50074?t=1639154575915. Published December 12, 2021.
31. Vouriot CVM, Burridge HC, Noakes CJ, Linden PF. Seasonal variation in airborne infection risk in schools due to changes in ventilation inferred from monitored carbon dioxide. *Indoor Air.* 2021;31(4):1154-1163.
32. Burridge HC, Bhagat RK, Stettler MEJ, et al. The ventilation of buildings and other mitigating measures for COVID-19: a focus on wintertime. *Proceedings of the Royal Society A: Mathematical, Physical and Engineering Sciences.* 2021;477(2247):20200855.
33. Lindsley WG, Derk RC, Coyle JP, et al. Efficacy of Portable Air Cleaners and Masking for Reducing Indoor Exposure to Simulated Exhaled SARS-CoV-2 Aerosols - United States, 2021. *MMWR Morb Mortal Wkly Rep.* 2021;70(27):972-976.
34. IHME Covid- Forecasting Team. Modeling COVID-19 scenarios for the United States. *Nat Med.* 2021;27(1):94-105.
35. Abaluck J, Kwong LH, Styczynski A, et al. Impact of community masking on COVID-19: A cluster-randomized trial in Bangladesh. *Science.* 2021:eabi9069.
36. Chu DK, Akl EA, Duda S, et al. Physical distancing, face masks, and eye protection to prevent person-to-person transmission of SARS-CoV-2 and COVID-19: a systematic review and meta-analysis. *Lancet.* 2020;395(10242):1973-1987.
37. Brooks JT, Butler JC. Effectiveness of Mask Wearing to Control Community Spread of SARS-CoV-2. *JAMA.* 2021;325(10):998-999.
38. Centers for Disease Control and Prevention. Science Brief: Community Use of Masks to Control the Spread of SARS-CoV-2. https://www.cdc.gov/coronavirus/2019-ncov/science/science-briefs/masking-science-sars-cov2.html. Updated December 6, 2021. Accessed December 13, 2021.
39. Clapp PW, Sickbert-Bennett EE, Samet JM, et al. Evaluation of Cloth Masks and Modified Procedure Masks as Personal Protective Equipment for the Public During the COVID-19 Pandemic. *JAMA Intern Med.* 2021;181(4):463-469.
40. Rothamer DA, Sanders S, Reindl D, Bertram TH. Strategies to minimize SARS-CoV-2 transmission in classroom settings: combined impacts of ventilation and mask effective filtration efficiency. *Science and Technology for the Built Environment.* 2021;27(9):1181-1203.
41. Centers for Disease Control and Prevention. Guidance for COVID-19 Prevention in K-12 Schools. https://www.cdc.gov/coronavirus/2019-ncov/community/schools-childcare/k-12-guidance.html. Updated July 9, 2021. Accessed July 16, 2021.
42. Centers for Disease Control and Prevention. Science Brief: Transmission of SARS-CoV-2 in K-12 Schools and Early Care and Education Programs – Updated. https://www.cdc.gov/coronavirus/2019-ncov/science/science-briefs/transmission_k_12_schools.html. Updated November 16, 2021. Accessed December 13, 2021.
43. Falk A, Benda A, Falk P, Steffen S, Wallace Z, Hoeg TB. COVID-19 Cases and Transmission in 17 K-12 Schools - Wood County, Wisconsin, August 31-November 29, 2020. *MMWR Morb Mortal Wkly Rep.* 2021;70(4):136-140.
44. Zimmerman KO, Brookhart MA, Kalu IC, Boutzoukas AE, McGann KA, Smith MJ, Maradiaga Panayotti GM, Armstrong SC, Weber DJ, Moorthy GS, Benjamin DK; ABC Science Collaborative. Community SARS-CoV-2 surge and within-school transmission. *Pediatrics*. 2021;148(4):e2021052686. doi:10.1542/peds.2021-052686
45. Jalal H, Dowd B, Sainfort F, Kuntz KM. Linear Regression Metamodeling as a Tool to Summarize and Present Simulation Model Results. *Medical Decision Making.* 2013;33(7):880-890.

© 2022 Giardina J et al. *JAMA Network Open.*
